# Supplementary material for: Social presence and dynamics of group communication: An analysis of a health professionals WhatsApp group chats
Source: PLoS One. 2023 Jul 17;18(7):e0288773. doi: 10.1371/journal.pone.0288773 (PMC10351686; doi:10.1371/journal.pone.0288773)
Supplement: S1 File — (DOCX) [file pone.0288773.s001.docx]

8:28 PM - **ANON 1**: *ACAPN keeps making headlines*

The above *press release* is a must read!

Long live ACAPN!

Long live physiotherapy in Nigeria!

8:32 PM – **ANON 2**: 💪💯

9:12 PM - **Anon 3**: Greater heights ma, @**Contact 1** congratulations🍾

9:12 PM - **Anon 3**: 👍🏽👍🏽

9:13 PM - **Anon 4**: This is good for us.

To God be the glory

9:13 PM - **Anon 4**: Thank you **P1**.

10:05 PM - **Anon 5**: Big congratulations ma

More wins i wish you

10:06 PM - **Anon 5**: This is goooooood! 💪🏾

7:13 AM - **Anon 6**: Good news👏👏👏

7:55 AM - **Anon 7**: Good development.

Kudos to the leader.

7:56 AM -**Anon 8**: That's great.

5:04 PM - **Anon 9**: It looks Like a Miracle But It is not _**P2**

http://opr.news/20349540201209en_ng?client=news

8:25 PM - **Anon 10**: This message was deleted

10:26 AM - **ANON 11**: <Media omitted>

12:02 PM - **Anon 12**: This message was deleted

12:02 PM - **Anon 12**: This message was deleted

12:02 PM - **Anon 12**: This message was deleted

3:44 PM - **ANON 1**: The Association of Clinical and Academic Physiotherapists of Nigeria (ACAPN) has honored **P3**,with an award of commendation for his outstanding role as distinguished xxxxxxx at the just concluded 4th/1st Virtual Scientific Conference 2020 as well as his contributions to the growth of ACAPN in the country.

ACAPN keeps making headlines! To get the full details, click on the link below:

https://s.docworkspace.com/d/AB4Fj-nt-stbqInn0qadFA

Long live ACAPN!

Long live Physiotherapy Profession in Nigeria!

10:55 AM - **Anon 13**: https://tribuneonlineng.com/physiotherapists-demand-revisit-of-legislation-protecting-persons-with-disabilities/ Physiotherapists demand revisit of legislation protecting persons with disabilities

11:03 AM - **Anon 14** added **P4**

9:47 AM - **Anon 15**: <Media omitted>

Do not carry over your ACAPN annual dues into the new year, make it a priority before the year ends. See above picture 👆👆👆for details

10:36 AM - **ANON 11**: <Media omitted>

Published by Saturday punch 12 Dec 2020

11:16 AM - **Anon 61**: This is great Sir. Welldone my Supervisor sir

12:35 PM - **Anon 6**: 👏👏👏

12:43 PM - **Anon 16**: Well done sir!

1:05 PM - **Anon 17**: <Media omitted>

Good afternoon colleagues. If you registered for this conference, it will be starting today at 2:30pm Nigerian time. Kindly join in.

5:21 PM - **Anon 15**: Good afternoon everyone.

The Nigerian Journal Of Physiotherapy and Rehabilitation volume 1 no1 is out as we are all aware that it was launched at the just concluded ACAPN virtual conference. The dispatch of this journal to only *up to date ACAPN financial members* would begin later this week. Thank you all.

ACAPN Xxxxxx.

5:21 PM - **Anon 15**: REMINDER TO PAY YOUR ANNUAL DUES

Are you yet to pay your annual dues?, please make it a priority as your dues are part of what drives the association forward.

BELOW IS THE CATEGORY OF PAYMENT

A. Interns/Corper- #5000

B. 1-10yrs post graduation- #7000

C. Above 10yrs of graduation- #10,000.

Pay to First Bank 2032549328. Association of Clinical and Academic Physiotherapists of Nigeria.

5:31 PM - **Anon 13**: Good

5:43 PM - **Anon 15**: Annual dues for **P10** received and acknowledged with thanks

8:48 PM -**Anon 8**: Noted.

1:12 PM - **ANON 1**: Highly esteemed colleagues,

The Nigerian Journal of Physiotherapy and Rehabilitation is the official publication of the Association of Clinical and Academic Physiotherapists of Nigeria (ACAPN). It aims at providing an avenue for researchers and practitioners from various specialties in physiotherapy and other rehabilitation-related disciplines to share their ideas and research findings, and foster the use of research in forming public policy.

Are you an author? Do you want to contribute to the journal? Click on the link below to get the details you need:

https://s.docworkspace.com/d/AKBMm8Dt-stbsPvN2qadFA

Long live ACAPN.

Long live Physiotherapy Profession in Nigeria!

10:39 PM - **Anon 17**: <Media omitted>

To register follow this link: https://forms.gle/BDrjJ5xVvagyjsqL7

6:24 AM - **ANON 11**: <Media omitted>

11:54 AM - **Anon 9**: Why I Begged My Husband To Get Another Wife - Housewife

http://opr.news/ac9a932201216en_ng?client=news

4:53 PM - **Anon 18**: <Media omitted>

6:53 AM - +**Anon 19** left

-**Anon 20** added **Contact 2**

3:36 PM - **Anon 4**: Good evening everyone.

I trust you are all keeping safe and healthy.

I want to reintroduce you all the international society for quality of life research, a global community of researchers, clinicians, healthcare professionals, consultants and patient research partners.

A society that allows for collaborations beyond physiotherapy. It is about creating a future in which patient perspective is integral to health research, care and policy.

I want to appreciate all our members who attended the virtual conference this year.

It is worthy of note that a minimum of six members presented at the conference.

That is a welcome development. I want to inform you that the developing nations special interest group was inaugurated at the conference. I want to enjoin all of you to be a part of this SIG in addition to the other SIGs.

The 2021 Annual Conference will be held 13-16 October 2021, in Calgary, Alberta, Canada.* Visit the conference website for further event details. isoqol.org

Watch the video and get to see me talk about how I joined ISOQOL

It is not too early to start preparing for the conference.

Let us go there and meet in Calgary.

God bless you

**P5**

Co-chair developing nations SIG

3:40 PM - **Anon 21**: Great, our own person is in the inner Caucasus. We got to be there. Well big sis 👍🏼👍🏼

4:18 PM - **Anon 22**: <Media omitted>

sticker8590349546347696017.wasticker_webp

8:26 PM - **ANON 1**: <Media omitted>

ACAPN holds Ordinary General Meeting (OGM)! Agenda include a Bye-Election for the office of the xxxxxx and Any other Business. See the attached documents for full details!

8:26 PM - **ANON 1**: <Media omitted>

ACAPN XXXXXXIAL ELECTION FORM 2020 -2021.pdf

8:44 PM - **ANON 1**: <Media omitted>

The Association of Clinical and Academic Physiotherapists of Nigeria (ACAPN) holds Ordinary General Meeting (OGM)!

Agenda include Bye-Election for the office of the xxxxxx and Any other Business. See the attached document for more details!

To get the nomination form for the election, click on the link below:

https://s.docworkspace.com/d/AIsqNGXt-stbiLmF46adFA

Long live ACAPN!

Long live Physiotherapy Profession in Nigeria!

10:25 PM - **Anon 6**: 👍👍👍

6:37 AM - **Contact 2**: Please can this be resend?

9:42 AM - **Anon 23**: https://docs.google.com/forms/d/1kuJeO61QfZFduj5X48YbYxQpnZziHUgJDr2IR8Iau-8/edit?usp=drivesdk

Dear Colleague

This questionnaire is simply to find out about your knowledge and practice of blood pressure measurements.

Kindly respond to the questions. Thank you

***P6***

2:00 AM - **Anon 24**: <Media omitted>

5:44 PM - **ANON 1**: <Media omitted>

The Association of Clinical and Academic Physiotherapists of Nigeria (ACAPN) holds Ordinary General Meeting (OGM)!

Agenda include Bye-Election for the office of the xxxxxx and Any other Business.

*Qualification*:

In line with Article 36.2 of ACAPN Constitution, the *xxxxxxial nominee* shall have graduated from any MRTB accredited Physiotherapy programme for a period of *not less than twelve (12) years*.

In the same vein, such individual must have registered with the association as a full member and *paid ACAPN dues up-to-date without any outstanding dues/levies* according to Article 36.1 of ACAPN Constitution.

To get the nomination form for the election, click on the link below:

https://s.docworkspace.com/d/AIsqNGXt-stbiLmF46adFA

Long live ACAPN!

Long live Physiotherapy Profession in Nigeria!

8:10 AM -**Anon 20**: <Media omitted>

8:11 AM - **Anon 15**: Same to you sir

8:18 AM - **Anon 25**: I wish you the same my boss

11:11 AM - **Anon 26**: Wish you the same Sir

11:22 AM - **Anon 27**: Wishing you the same Sir.

Thank you Sir

11:29 AM -**Anon 8**: Wish you the same sir.

11:39 AM - **Anon 28**: I wish you same sir

4:33 PM - **ANON 29**: My wishes also to you sir

7:14 PM - **Anon 15**: <Media omitted>

Reminder to pay your annual dues before the year ends

7:43 PM - **Anon 30**: Merry Christmas & Happy New year in advance. Keep faith alive. Regards to all our highly esteemed colleagues over there.

7:51 PM - **Anon 31**: Same to you Sir

7:51 PM - **Anon 32**: Wish the same sir

7:58 PM - **Anon 4**: Thank you Prof. A blissful season to us all.

8:02 PM - **Anon 33**: Thank you prof.

Seasons greetings

8:08 PM - **Anon 7**: 🙏🙏🙏 🍿 🍽️ 🍾🥂

8:27 PM - **Anon 34**: Thanks Prof.

We wish you a beautiful Christmas and happy new year in advance

1:00 AM - **ANON 1**: This message was deleted

1:01 AM - **ANON 1**: This message was deleted

1:01 AM - **ANON 1**: This message was deleted

1:08 AM - **ANON 1**: Same to you Prof.🍎🍐🥒🍾

1:12 AM - **ANON 1**: <Media omitted>

*SEASONS GREETINGS from the Association of Clinical and Academic Physiotherapists of Nigeria (ACAPN)*

7:46 AM - **Anon 35**: LAST AND FINAL BOARDING CALL

Good morning XXXXXXX ACAPN and other associates🧐 who registered for the first Physiotherapy Virtual Conference in the South of Sahara, ACAPN conference 2020 and your manifest is with me. Please, rush down to the gate for your souvenir in my office. There may be no opportunity for boarding after Christmas and definitely, no more boarding after ASUU strike is called off because I reserved the right to confiscate all unboarded souvenir.

Signed

Management, ACAPNXXXXXXX

7:59 AM - **Anon 30**: *I am **P7**.*

*Open this once* 👇👇

https://openit.site/en/f-chn?f=****

1:27 PM - **Anon 36**: Wishing you same!

11:51 PM - **Anon 38**: It's same here sir! Thanks, **Anon 116**.

10:57 AM - **ANON 1**: <Media omitted>

Wow! Christmas is less than 24 hours away! *Compliments of the season to you all from the Association of Clinical and Academic Physiotherapists of Nigeria (ACAPN)*. Happy celebrations!🍾🥒🍎🥃🍷🥥🍐🍋🍎💃🏻💃🏻💃🏻💃🏻

1:17 PM - **Anon 6**: 👏👏👏

2:11 PM - **Anon 4**: STK-20201224-WA0034.webp (file attached)

2:45 PM -**Anon 8**: 🙏

3:09 PM - **Anon 37**: 🌸 Hope i am the first person to send you this *wish message*

*Reply after viewing* 🌸

👇👇👇👇

>> https://wishenger.com/hsas/en/?f=O*****-

8:22 PM - **Anon 38**: <Media omitted>

8:22 PM - **Anon 38**: 👆🏽page 29, DAILY SUN newspaper of 23rd December, 2020.

6:29 AM - **Anon 31**: <Media omitted>

6:39 AM - **Anon 41**: <Media omitted>

9:17 AM - **ANON 1**: <Media omitted>

*MERRY CHRISTMAS* to you all from the Association of Clinical and Academic Physiotherapists of Nigeria (ACAPN). May this season bring you Joy, peace, success and blessings all the days of your life! Happy celebrations!

9:20 AM - **Anon 35**: <Media omitted>

10:04 AM - **Anon 25**: <Media omitted>

4:09 PM - **Anon 39**: STK-20201225-WA0041.webp (file attached)

5:37 PM - **Anon 40**: Merry Christmas to you all

6:16 PM - **Anon 9**: We gave 13,852 acres to Obafemi Awolowo University, not 11,961 hectares, says Ife community

http://opr.news/3965a259201222en_ng?client=news

6:35 AM - **Anon 17**: *Merry Christmas to us all*💕💕

As we celebrate and rest from the year's hustles, it's good to know that this is a good time to pull out that manuscript, dust it up and *submit for publication in the next issue of our dear ACAPN journal*.🙂

Please let our papers be represented in our own journal.🙏

*Charity begins at home*👍

7:38 AM - **ANON 1**: <Media omitted>

The Association of Clinical and Academic Physiotherapists of Nigeria (ACAPN) holds Ordinary General Meeting (OGM)!

Agenda include Bye-Election for the office of the xxxxxx and Any other Business.

*Qualification*:

In line with Article 36.2 of ACAPN Constitution, the *xxxxxxial nominee* shall have graduated from any MRTB accredited Physiotherapy programme for a period of *not less than twelve (12) years*.

In the same vein, such individual must have registered with the association as a full member and *paid ACAPN dues up-to-date without any outstanding dues/levies* according to Article 36.1 of ACAPN Constitution.

To get the nomination form for the election, click on the link below:

https://s.docworkspace.com/d/AIsqNGXt-stbiLmF46adFA

Long live ACAPN!

Long live Physiotherapy Profession in Nigeria!

7:43 AM - **ANON 1**: Highly esteemed colleagues,

The Nigerian Journal of Physiotherapy and Rehabilitation is the official publication of the Association of Clinical and Academic Physiotherapists of Nigeria (ACAPN). It aims at providing an avenue for researchers and practitioners from various specialties in physiotherapy and other rehabilitation-related disciplines to share their ideas and research findings, and foster the use of research in forming public policy.

Are you an author? Do you want to contribute to the journal? Click on the link below to get the details you need:

https://s.docworkspace.com/d/AKBMm8Dt-stbsPvN2qadFA

Long live ACAPN.

Long live Physiotherapy Profession in Nigeria!

9:22 AM - **ANON 1**: <Media omitted>

*ACAPN Presents plaque to special guest of honor*! The Association of Clinical and Academic Physiotherapists of Nigeria (ACAPN) has honored our special guest of honor in the just concluded 4th Scientific/Virtual conference, **P8** by presenting a wonderful plaque to him. This was done by our very own **P9**👏👏. Long live ACAPN. Hope you're all enjoying the season? Compliments of the season once more!🍾🍋🍎🍐💃🏻💃🏻💃🏻💃🏻🥃🍷🥒🍾

2:33 PM - **Anon 42**: <Media omitted>

11:48 PM - **Anon 4**: https://m.facebook.com/story.php?story_fbid=10225837579946359&id=1238480572&sfnsn=scwspwa

11:51 PM - **Anon 4**: Please watch and share the video of our presentations on disability and climate change from Nigeria

11:51 PM - **Anon 4**: I am a part of an organisation Sustain our abilities. Please watch, like my videos and subscribe to the channel. Thank you

11:51 PM - **Anon 4**: https://m.facebook.com/story.php?story_fbid=10225837474823731&id=1238480572&sfnsn=scwspwa

11:53 PM - **Anon 4**: https://m.facebook.com/story.php?story_fbid=3010664412288068&id=100000336169949&sfnsn=scwspwa

9:21 AM - **Anon 41**: Thanks so much for this!

4:16 PM - **Contact 3** left

12:12 AM - **ANON 1**: <Media omitted>

*ACAPN wishes you a Happy and Prosperous New year. Have a great year*!

12:21 AM - **ANON 1**: <Media omitted>

*Happy New year to you all. Excel in all you do*!

12:28 AM - **Anon 43**: <Media omitted>

12:32 AM - **Anon 31**: <Media omitted>

12:34 AM - **Anon 41**: This message was deleted

12:39 AM - **Anon 41**: <Media omitted>

12:46 AM - **Anon 41**: <Media omitted>

5:12 AM - **Anon 33**: Wishing us all a great and happy new year.

May 2021 bring us great tidings and all those good things that 2020 denied us in Jesus name Amen.

5:44 AM - **Anon 44**: Happy and prosperous new year. May the Lord bless you and your family and keep you. May He shed his face upon you and may nations come to know that He is your salvation. With love from the Akosiles.

7:23 AM - **Anon 25**: 🎁 *Hello* 🎁

🎁 *Hello* 🎁

🎁 *Hello* 🎁

💣💖 *Its a Surprise* 🎇🤗

*Open to see* 🤫🤭

‼️👇👇👇‼️

https://vejo.site/en/f-n2?f=********

9:05 AM - **Anon 35**: Happy New year. I pray that God Almighty shall make this year the best you've ever lived.

10:15 AM - **Anon 45**: Happy new year people

11:26 AM - **Anon 32**: <Media omitted>

1:15 PM - **Anon 39**: <Media omitted>

, 2:44 PM - **Anon 17**: <Media omitted>

The best time to start is Now. Give women's health Physiotherapists the opportunity to *catch them young*.🏋️‍♀️🏌️‍♀️🤸‍♀️🚴‍♀️

8:16 PM - **ANON 11**: <Media omitted>

10:54 AM - **ANON 1**: *ATTENTION*!

Happy new year to you all once again! ACAPN is simply the best!

As you all know, this year is loaded with lots of activities and great events in ACAPN. To achieve our collective goal, all hands must be on deck. As a result, we need all councils/directorates, specialty groups etc to quickly draw up their programmes/workshops/seminars etc for the year and send to the PRO ACAPN via WhatsApp here (**Contact 4**). If you prefer email, you can send to **Email 1**. We need the dates and topics (if possible speakers) for such events.

This will be compiled in addition to ACAPN central programmes and other regular events *in the form of an e-bulletin* which will be made available for everyone. What's the purpose? Simple: To keep you abreast of ACAPN programmes and activities for the year.

Let's plan from now till the end of the month (January 31) so that the e-bulletin can come out as early as possible.💃💃💃❤️❤️.

God bless you all!

Long live ACAPN!!

11:10 AM - **Anon 45**: Wonderful

11:11 AM - **ANON 1**: *ATTENTION*!

This is a crucial announcement!

The following ACAPN members have be appointed to serve in the following capacities:

1. *Scientific council*

Xxxxxxx: **P11**

Sccc: **P12**

**P13** - Member

*Publication council*👇

**P10** - Assistant Sccc.

Congratulations to you all. More grace to serve!

Long live ACAPN!💃🏻❤️❤️!

11:27 AM - **ANON 1**: Highly esteemed members of ACAPN,

I bring you greetings once again from the desk of ACAPN PR-CREW

ACAPN is committed to bringing you great and exciting events for your benefits and that of the Physiotherapy profession at large.👏👏 You know what? You can never go wrong with ACAPN.👏💃🏻💃🏻.

Follow us on Twitter @ACAPN4

Our website remains <http://www.acapn.org.ng>

Visit our website regularly for updates.

Long live ACAPN!

Long live Physiotherapy Profession in Nigeria!

PR-CREW, ACAPN.

1:27 PM - **Anon 46**: Congratulations on ur appointment.

More Grace.

Cheers🌹🙏

9:15 PM - **Anon 7**: Congratulations to them for qualities possessed to be fit for the job. More grace to them to serve more.

8:23 AM - **Anon 45**: Please I need a PT in Xxxxxxx- Ajah axis precisely for a PP. Inbox me please.

8:26 AM - **Anon 47**: I need PT in Abia state(Aba town) Text me if ur interested.

4:13 PM - **Anon 15**: This message was deleted

4:14 PM - **Anon 15**: This message was deleted

4:17 PM - **Anon 15**: 2021 Annual dues for **P14** received and acknowledged with thanks

4:17 PM - **Anon 15**: 2021 Annual dues for **P15** received and acknowledged with thanks

4:24 PM - **Anon 48**: <Media omitted>

Congratulations to Dr **P5**. Now promoted to be an Associate Professor. 🎉🎉🎉🎉🎉🎉🎉🎉

4:27 PM - **Anon 49**: Whaooooooo...Congratulations....Congratulations.... Congratulations...🙏🙏🙏🙏🙏🎼🎼🎼🎼👏👏👏👏👏

4:28 PM - **Anon 50**: Congratulations ma 🎉🎊

4:36 PM - **Anon 51**: Congratulations to you, Ma!

4:38 PM - **Anon 52**: Congratulations Ma, May our Good Lord continue to bless you and lift you higher

4:45 PM - **Anon 53**: Congratulations. This is the Lord doing.

4:47 PM - **Anon 42**: Chai! I thank God ooo. Baba God you too much 4 my systa waka ooo. Big big congratulations

4:51 PM - **Anon 15**: Congratulations ma, more winnings to come

4:57 PM - **Anon 54**: Congratulations "**P15**".

I really lack words to express how happy I am at the moment.

4:58 PM - **Anon 55**: Wow

I happiness never allow me say anything...

4:59 PM - **Anon 39**: Its long overdue😃🤝🏽congratulation ma.

5:09 PM - **Anon 56**: Congràtulation Associate Professor **P5**.

More grease... Polishing the chair to remove "Associate".

5:10 PM - **Anon 18**: Long overdue BIG CONGRATULATIONS.

5:10 PM - **Anon 18**: STK-20201221-WA0023.webp (file attached)

5:11 PM - **Anon 57**: Congratulations 🍾🥂. It's been long awaited. Greater heights by His grace

5:11 PM - **Anon 42**: Na u dey receive n acknowledge urself? Lol

5:24 PM - **Anon 17**: Woooow💃💃💃💃💃

Congratulations Prof

5:27 PM - **Anon 14**: Congratulations ma. May God continue to take you to greater heights.

5:34 PM - **P4**: Congratulations ma

5:37 PM - **Anon 58**: Yeeeeppppeèeeeeeee💃🏽💃🏽💃🏽💃🏽💃🏽💃🏽💃🏽💃🏽

Congratulations Ma

5:40 PM - **Anon 48**: Dr. **P5** of the Department of Physiotherapy promoted Reader with effect from October 2015*

More to come, watch out!

5:48 PM - **Anon 54**: STK-20200622-WA0019.webp (file attached)

5:48 PM - **Anon 54**: STK-20201105-WA0050.webp (file attached)

5:49 PM - **Anon 26**: Congratulations madam

5:52 PM - **Anon 59**: Congratulations Ma

5:59 PM - **Anon 6**: Wow!!!!. Great news.

What a way to start the year.

Congratulations ma

6:00 PM - **Anon 48**: STK-20201006-WA0010.webp (file attached)

6:07 PM - **Anon 60**: Congrats ma

6:25 PM - **Anon 9**: http://opr.news/4aff558c210104en_ng?client=newslite

6:35 PM - **Anon 61**: Congratulations Ma. Happy for u

6:52 PM - **Anon 101**: Congratulations Ma.

6:55 PM -**Anon 20**: Big congratulations **P99**. It's long overdue but we thank God for His faithfulness. We are waiting for the next one soon.

6:58 PM - **Anon 102**: Congratulations ma

6:59 PM - **Anon 59**: Mummy oooo what a great way to start the new year. What a great news. Mummy mi ooo 💃💃💃💃💃💃💃💃

7:00 PM - **Anon 34**: Congratulations Dr. ****.

7:00 PM - **Anon 59**: Exactly 2015 wow. Delayed but not denied it can only be God oo

7:34 PM - **ANON 1**: Congratulations to you ma. It's well deserved!

Greater heights ahead Ma!

Cheers!

7:45 PM - **Anon 41**: STK-20200624-WA0000.webp (file attached)

7:48 PM - **Anon 4**: Dearly beloved Colleagues (Senior and Junior),

Thank you for your prayers, calls and good wishes. God will surely reward your show of love.

I am grateful

I give God all the glory. He is ever faithful.

Shouts of rejoicing shall not cease from our tabernacles in Jesus name.

We await the others, trusting God for those expecting too.

Sincerely,

**P5**

7:55 PM - **Anon 15**: 😂😂😂😂. Na the work wey we dey do sir.

8:28 PM - **Anon 91**: Congratulations ma

8:33 PM - **Anon 7**: This is a sweet news that glads ones heart..

*Congratulations*

8:41 PM - **Anon 7**: Please don't jump protocol, I am the one to announce the payment of your annual due.😁😁

9:06 PM - **Anon 43**: Congratulations ma! Truly well deserved!!

9:10 PM - **Anon 9**: Congratulations to you ma

9:15 PM - **Anon 59**: Yes oo

9:16 PM - **Anon 15**: Noted sir 😂😂😂😂

9:17 PM - **Anon 59**: Amen!!

9:28 PM - **Anon 103**: Our own Associate Prof **P99**😁..... 2021 is starting on a good note....💃🏾💃🏾💃🏾

9:30 PM -**Anon 8**: Congratulations ma. A year of greater Glory.

9:33 PM - **Anon 3**: 🙌🏽🍾.... congrats ma,

9:41 PM - **Anon 69**: Congrats Mama,I celebrate with you this awesome height🙏🙏.

10:31 PM - **Anon 28**: Congratulations ma.💃💃

10:42 PM - **Anon 62**: Congratulations to you ma.

11:24 PM - **Anon 32**: Congratulations ma

11:50 PM - **Anon 104**: Congratulations ma

1:52 AM - **Anon 92**: Congratulations madam, I pray for your good health and God support.

9:28 AM - **Anon 4**: Good morning friends.

Thank you for the congratulatory messages. I am thankful.

Amen to the prayers.

You shall all be celebrated

9:47 AM - **Anon 46**: Congratulations Mama with a very broad & loving heart to us. This is just the beginning ma. Lots of love, Cheers❤️🌹

9:49 AM - **Anon 96**: This is so great a news!

10:33 AM - **Anon 24**: **P99**, iye tun nko. Oga, oga lagaju. Full Prof, knocking soon.👍 congratulations

10:42 AM - **Anon 4**: Thank you beloved. I am grateful.

God showed me mercy. God's mercy will speak for you all in this new year.

Thankful heart,

**P5**

11:37 AM - **Anon 58**: <Media omitted>

11:37 AM - **Anon 58**: Recorded message from MRTB Registrar on issues concerning issuance of licenses annually. Please listen and act accordingly to instructions .

11:41 AM - **Anon 72**: Congratulations Madam,you merit it.

11:57 AM - **ANON 105**: Congratulations ma

12:04 PM - **Anon 75**: This is the Lord's doing and it is MARVELLOUS in our eyes.

He makes all things beautiful in His time.

To Him alone be all the glory!

CONGRATULATIONS ma, we celebrate your success and WELL DESERVED PROMOTION.

🤗🤗🤗😘😘😘

4:42 PM - **Anon 4**: STK-20210107-WA0059.webp (file attached)

4:42 PM - **Anon 4**: STK-20201224-WA0034.webp (file attached)

4:42 PM - **Anon 4**: STK-20201114-WA0057.webp (file attached)

7:50 PM - **Anon 61**: Congratulations ma, you will continue to see the goodness of God👍👍👍

7:52 PM - **Anon 66**: Congratulations!

9:04 PM - **Anon 23**: Big CONGRATULATIONS!!!

1/8/21, 5:12 AM - **Anon 61**: 🍓🍏 *Morning Tea* 🥐☕

The Word for Today

08 January 2021

*Obstacles To Answered Prayer (4)*

‘If you remain in Me and My words remain in you, ask whatever you wish.’ John 15:7 NIV

The fourth obstacle to prayer is unscriptural prayers. Jesus said, ‘If you remain in Me and My words remain in you, ask whatever you wish, and it will be done for you.’ Those who live by the principles of God’s Word don’t pray to win the lottery, or for their horse to finish first in the Melbourne Cup. They know that those are unscriptural prayers God won’t honour.

When you pray, your request must line up with His will. After leading the children of Israel for forty years in the wilderness Moses naturally longed to enter the Promised Land and celebrate victory with them; to ‘go over and see the good land beyond the Jordan—that fine hill country and Lebanon.’ (Deuteronomy 3:25 NIV)

But in spite of his prayers it didn’t happen. Why? ‘Because… the Lord was angry… and would not listen… “That is enough,” the Lord said. “Do not speak to Me anymore about this matter. Go up to the top of Pisgah and look west and north and south and east. Look at the land with your own eyes, since you are not going to cross this Jordan.”’ (Deuteronomy 3:26–27 NIV)

Prayer isn’t some kind of talisman, or lucky charm, or religious formula you repeat to get the results you want. When your prayers line up with the Scriptures and are in harmony with God’s will, then will He answer them.

This truth may not be palatable or popular in today’s ‘feel good’ generation where everybody wants all the benefits of God’s Kingdom on their own terms.

If you’re serious about getting your prayers answered—read the fine print!

SoulFood: Ex 16–18, Matt 5:27–37, Ps 42:6–11, Pr 1:28–31

The Word for Today is authored by Bob and Debby Gass and published under licence from UCB International Copyright © 2021

5:22 AM - **Anon 5**: Big congratulations ma

🎊🎊🎊🎊🎊🎊🎊🍾🍷

12:10 PM - **Anon 117**: Congratulations ma.

10:31 AM - **Anon 33**: https://mujeresporafrica.es/6th-edition-science-by-woman/final-beneficiaries-6th-editon-scienes-by-women/

Kindly join me in congratulating one of our own, **P16** of the department of physiotherapy, who is one of the 19 recipients of this award across Africa and 3 in Nigeria of the 6th edition of the Science by Women Africa postdoctoral fellowship. Cheers

10:37 AM - **Anon 17**: Woow.

Congratulations Ma.🎈🎈🎈

Greater heights we pray.

10:40 AM - **Anon 61**: Congratulations Ma. MORE to come

11:04 AM - **Anon 4**: Physiotherapy in Nigeria is blessed of God.

Congrats **P16**.

Keep flourishing and glowing ma

11:07 AM - **Anon 37**: Great, **Anon 57**. Congratulations. God Almighty will promote you more and give you more opportunities.

11:18 AM - **Anon 6**: Congratulations ma

12:11 PM - **Anon 15**: Congratulations to you ma

12:31 PM - **Anon 43**: Congratulations ma

12:53 PM - **Anon 42**: Congrats **P17**

2:20 PM -**Anon 20**: Congratulations!!! More heights in Jesus' name

2:36 PM - **Anon 55**: Congratulations Ma

Cheers @**Contact 5**

2:53 PM - **Anon 34**: Congratulations ma

3:10 PM - **Anon 7**: Congratulation. I rejoice with her and the Physiotherapy family.

5:26 PM - **Anon 58**: Congratulations my Prof

6:19 PM - **Anon 59**: Congratulations to **Anon 57**, silent achiever. The very first Yoruba physio lady I met and mistook to be Igbo ooo.

8:03 PM - **Anon 49**: Congratulations...! Congratulations...!!

Congratulations.....!!!

**Anon 57**. More plumes to your cap. Really proud of you. Regards . God bless. 🙏

8:46 PM - **Anon 36**: Congratulations to **Anon 57**. God bless Physiotherapy, God bless ACAPN.

11:17 PM - **Anon 60**: Congratulations Ma. Greater height and more awards in Jesus Namem Amen!

7:26 AM - **Anon 57**: This message was deleted

7:29 AM - **Anon 57**: Dear wonderful Profs and colleagues. I wish to sincerely thank you for your kind wishes on this award which really is for our dear profession. Together we shall all move higher in Jesus name 🙏. Thank you!!!

7:51 AM - **Anon 42**: She is not Yoruba , **Anon 106**. She be PH woman ooo

10:33 AM - **Anon 46**: Congratulations Ma. Greater achievement by God's grace. Cheers🌹🙏

12:18 AM - **Anon 38**: Congratulations ma.

5:11 PM - **Anon 4**: Dear Colleagues,

I trust we are all keeping safe.

Can you kindly add my invite to a webinar on sexuality and spinal cord injury in your schedule for February 2021?

The webinar is scheduled for February 13 2021.

The link for registration is

https://www.eventbrite.com/e/better-sex-best-practices-in-sci-tickets-136047584989.

5:11 PM - **Anon 4**: <Media omitted>

7:23 PM - **Anon 30**: A big congratulation to you **Anon 107** for your new achievement. You will surely get to the ultimate height IJMN

12:59 PM - **Anon 14** added **Contact 6**

9:17 AM - **Anon 61**: Congratulations ma, wishing you many more success

9:52 AM - **Anon 57**: Thank you my dear sister 🙏

9:53 AM - **Anon 13**: Congratulations ma.

6:26 PM - **Anon 54**: Congratulations to my Supervisor and Mentor.

6:49 PM - **Anon 57**: Thank you🙏

7:04 PM - **Anon 57**: Thanks **P16**

7:18 PM - **Anon 62**: Congratulations to you ma.

11:39 AM - **Anon 4**: *What teachers should teach students about their names*

https://punchng.com/what-teachers-should-teach-students-about-their-names/

There is something strange about the way many people who are below 30 years introduce themselves in speech and in writing these days. During an interview for a job or other issues, if you ask the interviewees to introduce themselves, you would most likely hear something like: “My names are **P17**.”

The first strange issue is that of one person saying “my names are”, implying that the person has many personalities or identities. Only people with a shady character have many identities. The second issue is that of saying the surname first and saying the first name last, thereby confusing the listener.

The challenge these days is that when one hears or sees a name, one cannot easily know which is the first name or surname. For those whose names contain names that are regarded as traditional surnames, it may be easier to decipher which is the surname, but for some other people, it may not be easy. Imagine a young man whose name is **P18** or **P17 P19, P20, P21** introducing himself this way. For you to ascertain which is his first name or surname, you have to ask him to clar**Anon 106** that. But it is not all the time that someone has the opportunity to ask the owner of a name to make this type of distinction. That is why a standard way of writing one’s name was developed a long time.

When you write your name, which should come first: Surname or first name? The answer is evident. Your given name is called your first name. It should always come first.

Question: “What is your name?”

Answer: “My name is **P20**,” not “My name is **P21**.”

Your surname or family name is also called “last name,” because it is meant to come last. If you write a book or publish an article in a newspaper, your first name or initials come first while your surname or last name comes last.

Remember that you grew up hearing of **P22** (not **P23**), P24 (not P25), P26 (P27), P28 (not P29), P30 (not P31) P32 (not P33), P34 (not P35), P36 (not P37), P38 (not P40), P41 (not P42), etc.

However, when names are written alphabetically on a school list, bibliography, voter register, recruitment list, etc, the surname may be written first by those compiling the list, to make it easy to locate people’s names. But note that whenever the surname comes first, something MUST happen: a comma must come after the surname, or the surname must be in uppercase.

Example:

*Name1*

*Name2*

*Name3*

*Name3*

*Name4*

*Name5*

*Name6*

.

The trend among those who are under 30 to always introduce themselves or write their names with the surname first is queer. First name is called first name because it is meant to come first.

Based on which is your surname, someone will know how to address you officially. If you introduce yourself as *Name*, the person can then address you as *Name*, not *Name*. When only one name is used with your title, it has to be with your surname, not your first name *Names example*

Furthermore, on the issue of whether to say: “My name is *Name*” or “My names are Name”, let it be reiterated that each human being has only ONE name.

Please take a look at these two expressions:

*name?*

*name?* The first is the real name of the great footballer *name?* of Brazil. The second is the full name of the former xxxxxx of Congo when it was known as *name?*. However, in spite of the length of the names, each of them is ONE name. *name?* has ONE name and *name?* has ONE name.

You should never ask someone: “What are your names?” And you should never tell someone: “My names are….” You are one person, not two or more people.

Yes, like most people, you may have a number of identifiers under your name, but they all form a unit known as your name. You can break them down into first name (or given name), middle name (second name), third name, fourth name, and surname (or family name or last name). But you only have ONE name and nothing more.

Some factors have made this issue of writing the surname first prevalent now. The first reason is the practice of emphasising the surname in schools. It has always been the practice that teachers address their students by their surname: “*name?*, come here. Where is *name?*

?” But based on the feedback I have received from my probe into the matter, many teachers these names specifically teach their students to introduce themselves with their surname first.

The second reason is that even when teachers have not expressly told their students to always write their surname first, they have not clearly made the students to understand that outside the school environment, they should introduce themselves first with their first name. There is no distinction made about how one should write one’s name when filling a form and when introducing oneself.

There is also the factor of the rise of the Internet. There is a rise in the number of places where one can fill one’s details online. There are email accounts to create, social media accounts to create, and different websites to register with for one thing or the other. In addition, the banks also lay emphasis on surnames in the opening of accounts and issuing of the ATM cards. Agencies that provide identification documents like the passport, driving licence, voter card, national identity card and company’s identification cards lay emphasis on the surname too.

However, in spite of all the challenges in the environment on the issue of the order of names, the first name still remains the first name, while the surname still remains the surname. If any institution requires the surname to be written first while filling a form, give it to them in that format. But do not let that confuse you to introduce yourself or have your name displayed on your social media platforms with your surname before your first name, unless you clearly mark the surname off with a comma. That is the standard way. In addition, even though you may have a first name, a middle name and surname, you have only one name. Therefore, you should not ask someone: “What are your names?” or introduce yourself with: “My names are ….”

These issues should be taken seriously by teachers from the primary school to the university, because when it comes to learning, teachers wield the biggest influence on children. To most children, whatever their teachers say is right is what is right.

11:49 AM - **Anon 63**: Very educative.

Thank you ma

1:24 PM - **Anon 7**: Thank you ma.

I knew the above late though, I am now well informed.

2:21 PM - **Anon 13**: <Media omitted>

Determinants of Lung function.pdf

2:32 PM - **Anon 61**: This is a great read. Thanks ma

2:32 PM - **Anon 61**: Welldone Sir.

3:55 PM - **ANON 64**: 👏👏👏👏

7:11 PM - **ANON 1**: <Media omitted>

AN ICON WAS BORN TODAY!

There are people whose excellent deeds continue to speak volumes even years after their death. Such is the case of this Icon of Physiotherapy profession who left this planet for the great beyond on April 29, 2016.

She was born on this day January 15, 1953. Her numerous impacts in Nigeria and beyond cannot be over emphasized. This Physiotherapy Icon was the first female xxxxxx of the Nigeria Society of Physiotherapy (2004 - 2008) and her impacts within the Physiotherapy profession and beyond still speak volumes.

She was a member of an array of professional organizations including the Health and Care Professional Council in UK, International Council for Health Education, Recreation, Sports and Dance, USA, World Confederation for Physical Therapy etc. She was a clinician, an educator and researcher with several award winning publications in peered reviewed journals and papers presented at both national and international conferences!

Words will fail us to actually describe this legend of many parts. She was a selfless and visionary leader, an excellent mother to all of us and a great wife to her husband. She was a believer in Christ indeed.

Her name is **P44**, PT of blessed memory. She would have been 68 years old today. Our great Mama, we the Association of Clinical and Academic Physiotherapists of Nigeria (ACAPN) celebrate and will continue to celebrate you even though you're no more!

Continue to rest in peace Ma.

PR-CREW, ACAPN.

7:15 PM - **Anon 15**: May her soul continue to rest in perfect peace

7:40 PM - **ANON 64**: 👏👏👏👏

7:51 PM - **Anon 59**: Amen

8:06 PM - **Anon 35** added **Contact 7**

8:08 PM - **Anon 35**: Good evening everyone. Please, indulge me to change my number of this platform

Thank you

8:08 PM - **Anon 35** left

8:11 PM - **Anon 41**: May her beautiful soul continue to rest peacefully.

11:46 PM - **Anon 61**: She will always be remembered

6:12 AM - **Anon 61**: May her soul continue to rest in the bosom of the Lord

10:10 AM - **Anon 32**: Rest in peace

12:16 PM - **Anon 45**: Rest on ma

5:42 PM - **Anon 13**: <Media omitted>

MSK pain among OT and PT students at UKZN.pdf

5:45 PM - **Anon 61**: Welldone Sir

8:49 PM - **Anon 46**: More Grace Sir

Cheers🌹🙏

8:53 PM - **Anon 4**: STK-20200327-WA0001.webp (file attached)

11:09 PM - **Anon 65**: STK-20200804-WA0010.webp (file attached)

12:25 AM - **Anon 55**: Well done Sir

👍👍👍

12:28 AM - **Anon 55**: Thank you for sharing the full text.

10:50 PM - **Anon 7**: There are few vacancies for Physiotherapy internship. Inform interested PTs to reach me.

10:58 PM - **Anon 5**: Interns are even scarce these time...when last did any school graduate interns 🤷🏾‍♂️

11:04 AM - **Anon 17**: <Media omitted>

The year has just started.

We are not waiting for it to start fully before we start learning.🌀

*ACAPN Women's Health Specialty Group* is inviting you for a free webinar (find details above).

It promises to be educative.👍👍

*See you in class*.🙂

11:24 AM - **Anon 6**: Hmmm, the year never start, Women's Health Speciality Group is about "intimidating" us again, just like last year🤣🤣🤣🤣.

Other Speciality Groups, over to you.

12:10 PM - **ANON 1**: 👏👏👏👏👏

12:12 PM - **ANON 1**: *ATTENTION*!

Happy new year to you all once again! ACAPN is simply the best!

As you all know, this year is loaded with lots of activities and great events in ACAPN. To achieve our collective goal, all hands must be on deck. As a result, we need all councils/directorates, specialty groups etc to quickly draw up their programmes/workshops/seminars etc for the year and send to the PRO ACAPN via WhatsApp here (**Contact 4**). If you prefer email, you can send to **Email 1**. We need the dates and topics (if possible speakers) for such events.

This will be compiled in addition to ACAPN central programmes and other regular events *in the form of an e-bulletin* which will be made available for everyone. What's the purpose? Simple: To keep you abreast of ACAPN programmes and activities for the year.

Let's plan from now till the end of the month (January 31) so that the e-bulletin can come out as early as possible.💃💃💃❤️❤️.

God bless you all!

Long live ACAPN!!

12:19 PM - **ANON 1**: <Media omitted>

The Association of Clinical and Academic Physiotherapists of Nigeria (ACAPN) holds Ordinary General Meeting (OGM)!

Agenda include Bye-Election for the office of the xxxxxx and Any other Business.

*Qualification*:

In line with Article 36.2 of ACAPN Constitution, the *xxxxxxial nominee* shall have graduated from any MRTB accredited Physiotherapy programme for a period of *not less than twelve (12) years*.

In the same vein, such individual must have registered with the association as a full member and *paid ACAPN dues up-to-date without any outstanding dues/levies* according to Article 36.1 of ACAPN Constitution.

To get the nomination form for the election, click on the link below:

https://s.docworkspace.com/d/AIsqNGXt-stbiLmF46adFA

Long live ACAPN!

Long live Physiotherapy Profession in Nigeria!

1:10 PM - **Anon 46**: 🌹🙏

1:46 PM - **Anon 58**: STK-20201112-WA0014.webp (file attached)

2:18 PM - **Anon 17**: This message was deleted

2:18 PM - **Anon 17**: 👍👍

5:03 PM - **Anon 66**: **P45** well done o!

5:26 PM - **Anon 17**: 😘

6:17 PM - **ANON 1**: *ATTENTION!*

This is a very important announcement!

The Association of Clinical and Academic Physiotherapists of Nigeria (ACAPN) wishes to ask members who have interest in community-based Physiotherapy to indicate as *ACAPN wants to launch community-based Physiotherapy Specialty group this year*.

Interested persons should contact the acting xxxxxx of ACAPN, **P46** via **Contact 7** immediately.

God bless ACAPN!

8:54 PM - **Anon 67**: 👍

10:30 PM - **Anon 5**: 👍🏾

7:13 AM - **Anon 68**: This message was deleted

12:03 PM - **Anon 17**: Wed, 27 Jan at 7:00 PM-Wed, 8 Oct at 11:00 AM GMT-05:00 Online event https://fb.me/e/3siZ48jm1?ti=wa

1/28/21, 12:58 PM - **Anon 47**: I cannot laugh this alone...

N100 and N1000 were best friends. One day they had an accident and both died. On reaching paradise entrance, Angel allowed N100 to enter freely. So N 1000 asked. My Lord, am I not 10 times more valuable than him? How can he enter while I'm outside? Angel answered. N100 & his siblings N50, N20, N10 always come to Church and even during Bible studies and prayer meetings, including midweek services but you are always present in parties, restaurants & shopping centre, so disappear, I don't want to see you.

Pls let's help N1000 to enter Heaven....take him to Church

Sir/ Ma N1000 is not too much for God sake. Abeg make we try dey put am inside offering boxes

Don't read alone,

*PASS IT ON!*

1:09 PM - **Anon 17**: <Media omitted>

🚶‍♀️ The 3 meter backwards walk test: a novel measure of fall risk walking.

❓ Do you use this test in your daily practice when working with those at risk of falling? It may be something to consider.

➡️ This brilliant graphic was made by Kevin Wernli Physio, and is based off a research paper we summarised.

📲 Our goal is to make it easier for you to understand and apply the latest and Physio research.

🔗 Learn more here https://www.physio-network.com/research-reviews/

3:22 PM - **Anon 108**: BREAKING NEWS!!?

**P47** SIGNS CORONAVIRUS DISEASE(COVID-19) HEALTH PROTECTION REGULATIONS 2021

SIGNED ON 26TH JANUARY, 2021

SEE CONTENTS BELOW: CORONAVIRUS DISEASE (COVID-19) HEALTH PROTECTION REGULATIONS 2021

In the exercise of the powers conferred upon me by Section 4 of the Quarantine Act, Cap. Q2 Laws of the Federation of Nigeria 2010 and all other powers enabling me in that behalf; and in consideration of the urgent need to protect the health and wellbeing of Nigerians in the face of the widespread and rising numbers of COVID-19 cases in Nigeria, I, **P47**, Xxxxxx of the Federal Republic of Nigeria, hereby make the following Regulations –

PART 1

Restrictions on Gatherings

1. At all gatherings, a physical distance of at least two metres shall be maintained at all times between persons.

2. Notwithstanding the provision of Regulation 1, no gathering of more than 50 persons shall hold in an enclosed space, except for religious purposes, in which case the gathering shall not exceed 50% capacity of the space.

3. All persons in public gatherings, whether in enclosed or open spaces, shall adhere to the provisions of Part 2 of these Regulations.

4. The provisions of these Regulations may be varied by Guidelines and Protocols as may be issued, from time to time, by the Xxxxxxial Task Force (PTF) on Covid-19 on the recommendation of the Nigeria Centre for Disease Control (NCDC).

PART 2

Operations of Public Places

Markets (including open markets), Malls, Supermarkets, Shops, Restaurants, Hotels, Event Centres, Gardens, Leisure Parks, Recreation Centres, Motor Parks, Fitness Centres, etc.

5. No person shall be allowed within the premises of a market, mall, supermarket, shop, restaurants, hotels, event centres, gardens, leisure parks, recreation centres, motor parks, fitness centre or any other similar establishment (hereinafter collectively referred to as “establishments”) except:

a. he is wearing a face covering that covers the nose and mouth;

b. he washes his hands or cleaned the hands using hand sanitiser approved by the National Agency for Food and Drug Administration and Control (NAFDAC); and

c. his body temperature has been checked. Any person found to have a body temperature above 38 degrees Celsius shall be denied entry and advised to immediately seek medical attention.

6. Every establishment occupier shall make provision for regular hand hygiene for any person coming into the premises during opening hours. This includes a handwashing station with soap and running water, or hand sanitiser approved by NAFDAC.

7. Each establishment occupier is responsible for cleaning and disinfecting his premises.

8. It is the responsibility of the occupier of the establishment to:

a. Enforce the proper use of face covering;

b. Make provision for safe hygiene facilities;

c. Enforce provision of temperature checks prior to entry into the establishment;

d. Enforce provisions and use of face covering within the establishment;

e. Ensure that their customers queue up and are attended to serially while complying with physical distancing measures and avoiding overcrowding; and

f. Generally, ensure compliance with the provisions of these Regulations.

9. Occupiers of establishments are liable for any observed non-compliance by any persons within the premises.

Places of Worship

10. All worshippers and users of places of worship shall comply with the provisions of Parts 1 and 2 of these Regulations.

11. Worshippers shall, where and whenever possible, avoid sharing worship items such as mats, bottles, hymnals, etc.

12. It is the responsibility of the person in charge of a place of worship to ensure compliance with the provisions of these Regulations within the worship centre.

Workplace and Schools

13. All persons entering the premises of a workplace or school shall comply with the provisions of Parts 1 and 2 of these Regulations.

14. It is the responsibility of the person in charge of a workplace or a school to ensure compliance with the provisions of these Regulations within the office or school premises.

Banks

15. All employees, customers and visitors of banks shall comply with the provisions of Parts 1 and 2 of these Regulations.

16. All banks shall comply with the guidelines, mode and scope of operations issued by the Central Bank and Federal Ministry of Finance as it relates to the COVID-19 response.

17. All banks shall develop a schedule for regular cleaning of buttons and surfaces of Mantrap Entrance Doors, ATM machines and other commonly used areas.

Public Transportation Vehicles

18. All operators and passengers of public transportation vehicles (hereinafter referred to as “operators”) shall comply with the provisions of Part 2 of these Regulations.

19. Operators shall ensure adequate spacing in between passengers.

20. Operators shall ensure frequent cleaning and disinfection of parts of the vehicle frequently handled by passengers and drivers such as doors and window handles/buttons, steering wheels and dashboards.

21. Operators shall encourage passengers to frequently perform hand hygiene.

22. All operators of trains, ships and planes shall have an occupational health and infectious diseases preparedness plan, in case an employee or traveller becomes unwell in the course of a journey.

23. It is the responsibility of operators to ensure compliance with the provisions of these Regulations in the course of their operations.

Hostels, Boarding Houses and Detention Centres

24. Managers of Hostels, Boarding Houses, Nursing Homes, Correctional Centres, Remand Homes, Holding Cells, Military Detention Facilities, and such centres for care and custody of persons, shall ensure compliance with the provisions of these Regulations.

25. Managers of such facilities shall ensure that suspected cases of Covid-19 are promptly and appropriately separated from others and are reported to medical officers of the State Ministry of Health for necessary action.

PART 3

Mandatory Compliance with Treatment Protocols

26. The health and safety protocols and guidelines are issued by the PTF on Covid-19 on the recommendation of the NCDC and shall be binding on all persons.

27. Persons confirmed to have tested positive to COVID-19 by an NCDC accredited laboratory, may not refuse isolation and or admission to a designated health establishment for management of the disease.

28. All public secondary and tertiary health facilities shall designate a space or holding bay for the initial triage or assessment of suspected persons with COVID-19 in line with the approved protocol for case management.

29. All public secondary and tertiary health facilities shall establish sample collection centres, where test samples from suspected cases can be collected and transmitted to an accredited testing laboratory in the State.

30. Notwithstanding the provision of Regulation 27, where a person confirmed to have tested positive to the COVID-19 is:

a. Asymptomatic or has mild symptoms; and

b. Proves able to make an alternative isolation arrangement that satisfies protocols issued by the NCDC, as certified by a healthcare provider and assessment by a medical professional;

such person may use such alternative arrangements, including supervised “home-based care,” for self-isolation and adhere strictly to the guidelines, until confirmed by the healthcare provider to be no longer at risk of infecting others with the virus.

PART 4

Offences and Penalties

31. Any person who contravenes the provisions of these Regulations commits an offence.

32. Any person who, without reasonable cause, contravenes a direction given under Parts 1 and 2 of these Regulations commits an offence.

33. Any person who, without reasonable cause, obstructs an authorised official from enforcement of these Regulations commits an offence.

34. An offence under these Regulations is punishable, on summary conviction, by a fine or a term of six months imprisonment or both in accordance with Section 5 of the Quarantine Act.

PART 5

Enforcement and Application

35. Personnel of the Nigeria Police Force, the Nigeria Security and Civil Defence Corps, the Federal Road Safety Corps, the Nigeria Immigration Service, the Federal Airport Authority of Nigeria, and other relevant Local Government, State and Federal Government agencies are hereby directed to enforce the provisions of these Regulations.

36. Any officer of the enforcement agencies who fails, neglects, or refuses to enforce the provisions of these Regulations shall be subject to disciplinary action by the disciplinary body of his respective agency.

37. The provisions of these Regulations shall apply throughout the Federal Republic of Nigeria.

38. State Governors may issue Regulations on further steps as may be considered necessary.

PART 6

Interpretation and Citation

39. In these Regulations, unless the context otherwise requires:

a. “Offence” means any act, which may constitute a violation of the provisions of these Regulations.

b. “Enforcement Agency” means any law enforcement or security agency vested with the statutory power to investigate and prosecute any person in respect of any of the applicable offences.

c. “Face covering” means a covering of any type (other than a face shield) which covers, fully, a person’s nose and mouth.

d. “gathering(s)” means an assembly or meeting of people.

e. “occupier” means any person who is in current occupation and control of premises either as an owner or lessee.

f. “Public transportation vehicle” means bicycle, motorcycle, tricycle, car, taxi, limousine, bus, train, ship, plane, or any other vehicle of transportation that carries more than one person at a time.

g. “reasonable cause” includes medical emergency, wherein the person in violation forgot to put on face covering, but complied with the directive to do so upon prompting; a natural disaster, wherein persons need to be evacuated into a public space; etc.

40. These Regulations shall take effect immediately and remain in effect until otherwise determined.

41. All other Protocols and Guidelines issued by the PTF, NCDC, and or State Governments, except as expressly provided, shall remain in force.

42. These Regulations may be cited as the Coronavirus Disease (Covid-19) Health Protection Regulations 2021.

12:09 PM - **ANON 1**: *ATTENTION*!

*Update on the appointment of ACAPN members into the Scientific council*

Xxxxxxx: **P15**

Sccc: **P16**

**P48** - Member

**P10** - Member

**P7** - Member

**P17** - Member

Congratulations to you all. More grace to serve!

Long live ACAPN!💃🏻❤️💃🤝

1:13 PM - **Anon 39**: 👍🏽

3:26 PM - **ANON 1**: *ATTENTION*!

This is a very important announcement!

**P49**. Ativie is now the new leader of the ACAPN Cardiopulmonary Specialty group.

Congratulations to you our wonderful IPPRO.

More power to your elbow!

Long live ACAPN!

💃💃❤️❤️🤝

1/29/21, 3:52 PM - **Anon 17**: <Media omitted>

Hello.

Just click the link below to join on us tomorrow.

http://meet.google.com/bhe-dasg-ykr

No registration is required.

6:08 PM - **Anon 39**: 💪🏽

2:02 PM - **ANON 1**: *Call for submission of Articles*!

We hereby call for submission of articles for the *3rd edition of our Absolute Physiotherapy Magazine*.

Absolute Physiotherapy Magazine is the official Magazine of the Association of Clinical and Academic Physiotherapists of Nigeria (ACAPN). We want to receive good articles from members of ACAPN on different areas. For example, one of our outline is *news* which could either be local, national or international! Of course these will be current/interesting physiotherapy news. We want to have them in our magazine!

Furthermore, we also have another section we call *features*! This area showcases different scientific articles on different areas of Physiotherapy. For example, we had a caption like "challenges of Women's Health and Physiotherapy education: clinical and academic perspective" in the last edition.

Therefore, the magazine is not only the affairs of the PR-CREW members alone. It's an ACAPN affair! That's why we are calling for articles from our members who are able to make their own contributions as well. It will make the magazine better!

In order to get the magazine produced on time, we are giving from now to the end of February 2021 for us to submit our articles. All articles should be submitted to the ACAPN PRO via email (**Email 1**).

Thank you as you join to make ACAPN better and also rebrand the Physiotherapy profession!

Long live ACAPN!

PR-CREW, ACAPN.

2:13 PM - **ANON 1**: *ATTENTION*!

This is an important announcement!

**P50** has been appointed as the new xxxxxxx of Conferences and Internalisation Council.

Congratulations to you Sir🤝🤝.

More power to your elbow!

Long live ACAPN!

💃💃❤️🤝!

2:14 PM - **Anon 17**: Hello.

This is starting off in a few minutes

1:52 PM - **ANON 1**: *ATTENTION*!

Our Ordinary General Meeting (OGM) is one month and twelve days away. It's *13th of March, 2021 to be precise*!

Please, prepare to participate!

Long live ACAPN!

1:53 PM - **ANON 1**: <Media omitted>

The Association of Clinical and Academic Physiotherapists of Nigeria (ACAPN) holds Ordinary General Meeting (OGM)!

Agenda include Bye-Election for the office of the xxxxxx and Any other Business.

*Qualification*:

In line with Article 36.2 of ACAPN Constitution, the *xxxxxxial nominee* shall have graduated from any MRTB accredited Physiotherapy programme for a period of *not less than twelve (12) years*.

In the same vein, such individual must have registered with the association as a full member and *paid ACAPN dues up-to-date without any outstanding dues/levies* according to Article 36.1 of ACAPN Constitution.

To get the nomination form for the election, click on the link below:

https://s.docworkspace.com/d/AIsqNGXt-stbiLmF46adFA

Long live ACAPN!

Long live Physiotherapy Profession in Nigeria!

2:09 PM - **ANON 1**: This message was deleted

2:12 PM - **ANON 1**: Don't forget! *March 13, 2021* is the day. Don't miss out of the OGM!

6:43 AM - **Anon 69**: Congratulations!!!!

6:45 AM - **Anon 69**: Congratulations to our proactive leader Dr Ativie

6:46 AM - **Anon 69**: Thanks for accepting this responsibility,Sir.

2:55 PM - **Contact 7**: Topic: Inaugural Lecture of **P51**

Time: Feb 17, 2021 04:00 PM West Central Africa

Zoom Meeting Details:

https://wacren.zoom.us/j/62774543277?pwd=MXIwTlp1T1AwWHVFYzVHMGc3d0pndz09

Meeting ID: 627 7454 3277

Passcode: 463013

6:29 PM - **Anon 58**: STK-20201115-WA0127.webp (file attached)

6:55 PM - **Anon 61**: 👏👏👏

8:34 PM - **Anon 41**: STK-20200522-WA0017.webp (file attached)

5:11 PM - **Anon 15**: <Media omitted>

Please send me a personal message when this is done. ACAPN Xxxxxx

3:55 PM - **Anon 101**: ***twitter 1***

7:57 PM - **Anon 15**: Annual dues for **Anon 57** received and acknowledged with thanks

9:23 PM - **Anon 70**: <Media omitted>

12:36 PM - **Anon 17**: *Good morning colleagues*

Is there any of us that got infected with the covid-19 virus❓

Please private chat me urgently if you are involved.

**P46**

3:24 PM - **Anon 71**: STK-20200616-WA0026.webp (file attached)

3:55 PM - **Anon 72**: This message was deleted

3:57 PM - **Anon 72**: This message was deleted

9:41 AM - **ANON 1**: *REMINDER*!

Our Ordinary General Meeting (OGM) is one month and three days away. It's *13th of March, 2021 to be precise*!

Please, prepare to participate!

Remember that *nomination closes on 20th February, 2021* which is three weeks before the OGM!

If you have not downloaded and filled the nomination form, please do it now!

Long live ACAPN!

9:43 AM - **ANON 1**: <Media omitted>

The Association of Clinical and Academic Physiotherapists of Nigeria (ACAPN) holds Ordinary General Meeting (OGM)!

Agenda include Bye-Election for the office of the xxxxxx and Any other Business.

*Qualification*:

In line with Article 36.2 of ACAPN Constitution, the *xxxxxxial nominee* shall have graduated from any MRTB accredited Physiotherapy programme for a period of *not less than twelve (12) years*.

In the same vein, such individual must have registered with the association as a full member and *paid ACAPN dues up-to-date without any outstanding dues/levies* according to Article 36.1 of ACAPN Constitution.

To get the nomination form for the election, click on the link below:

https://s.docworkspace.com/d/AIsqNGXt-stbiLmF46adFA

Long live ACAPN!

Long live Physiotherapy Profession in Nigeria!

5:40 AM - **Anon 73**: If you know anybody by the name **P51** who graduated from the University of Ibadan. (Yoruba)

All her original certificates like certificate of origin (Ibadan north east), birth certificate, primary school cert, National ID card, and secondary school certificate were found by the roadside in Ibadan . Call **Contact 9**

Pls help us to share on all WhatsApp group page. God bless u

11:15 PM - **Anon 17**: ⭕⭕ *Good evening Esteemed colleagues*⭕⭕

Will you be interested in attending a paid workshop on *Physiotherapy management of erectile dysfunctions*?

If yes, kindly private chat me before the end of 12/2/21.

Thanks

7:02 PM - **Anon 4**: Dear Friends, colleagues and individuals living with disabilities, kindly register for the global webinar through this link.

Time is 3pm Nigerian time

Saturday, February 13, 2021.

Thank you

https://www.eventbrite.com/e/better-sex-best-practices-in-sci-tickets-136047584989

7:02 PM - **Anon 4**: <Media omitted>

11:09 PM - **Anon 17**: *ATTENTION!*

This is a very important announcement!

The Association of Clinical and Academic Physiotherapists of Nigeria (ACAPN) wishes to ask members who have interest in community-based Physiotherapy to indicate as *ACAPN wants to launch community-based Physiotherapy Specialty group this year*.

Interested persons should contact the acting xxxxxx of ACAPN, **P46** via **Contact 7** immediately.

God bless ACAPN!

11:42 PM - **ANON 11**: <Media omitted>

12:01 AM - **ANON 11**: <Media omitted>

2:05 AM - **ANON 1**: <Media omitted>

The Consortium for Advanced Leadership and Mentorship Programme (CALMP) of the Association of Clinical and Academic Physiotherapists of Nigeria (ACAPN) is here again!

The importance of Leadership and Mentorship in our society today cannot be over emphasized. It is a well known fact that the success of every organization and the society at large depends largely on leadership. When people are mentored properly, they become great leaders!

You've got to seize this great and life transforming opportunity! Get informed, trained by great resource persons and be transformed!

See the fliers for the full details of the programme!

Long live ACAPN!

2:05 AM - **ANON 1**: <Media omitted>

3:28 PM - **Anon 4**: Eventbrite

Hi everyone,

Our webinar “Better Sex” will begin shortly. Please join us at our youtube channel:

https://www.youtube.com/channel/UCqtL5Ua105tqD-dxwyODdsw

Don't forget to subscribe to the channel!

See you there!

The Sustain Our Abilities Team

3:29 PM - **Anon 4**: Thank you my dear

Please go to YouTube sustain our abilities channel.

My presentation is just for 10 minutes. Rounding off in few minutes.

Thank you

4:59 PM - **Anon 4**: Kindly join

https://youtu.be/n37tmulofGQ

For the Question and Answer session

5:38 PM - **Anon 59**: You can still join in

7:28 PM - **Anon 4**: Thank you for joining. You can watch the video via this link

https://youtu.be/n37tmulofGQ

7:36 PM - **Anon 9**: Ok, good

10:23 PM - **Anon 36**: https://www.businessinsider.in/science/health/news/regular-physical-activity-reduces-instant-death-risk-from-heart-attack-says-new-study/articleshow/80897478.cms

12:50 PM - **Anon 74**: This message was deleted

3:47 PM - **ANON 1**: This message was deleted

3:57 PM - **ANON 1**: <Media omitted>

Dear colleagues,

This is another opportunity for you to update yourself. Ogun State chapter of ACAPN brings you an exciting webinar captioned "Knowledge Translation for Physical Rehabilitation Specialist"!

You can't afford to miss it. Knowledge is power. See the flier for more details!

4:00 PM - **Anon 28**: STK-20210215-WA0024.webp (file attached)

5:19 PM - **Anon 6**: 👍👍👍

6:18 PM - **Anon 10**: This message was deleted

10:28 AM - **Anon 61**: 👏👏👏

3:57 PM - **Anon 15**: 2021 Annual dues for **Anon 33** received and acknowledged with thanks

6:15 PM - **Anon 15**: 2021 annual dues for **P52** received and acknowledged with thanks

10:35 PM - **Anon 17**: <Media omitted>

APGR-24-2-4.pdf

10:56 PM - **Anon 46**: Congratulations Ma🙏🌹

6:04 AM - **Anon 42**: Gr8 job

10:34 PM - **ANON 11**: <Media omitted>

NUC..pdf

10:35 PM - **Anon 24**: <Media omitted>

CIRCULAR. 19TH JANUARY 2021_0002.jpg

10:35 PM - **Anon 24**: <Media omitted>

CIRCULAR. 19TH JANUARY 2021_0001.jpg

8:20 AM - **Anon 7**: Thank God for this development. I had been expecting it before now after the stakeholders meeting called by the MRTB on the approval given by the NUC in 2018.

Our colleagues in the Academics should please work harder to see to early commencement of the DPT and the transition of the current B.Sc to DPT.

We should not forget that our friends in the medics are not well dispose to it. Even though they not stop it, however, delay may not be favourable.

Once again I want to thank all that God used to get it done.

This is collective efforts of all the Physiotherapists; starting from the MRTB, the academia and the clinicians.

Please, the young and the younger ones should not relent though, I may not belong to that class. The seniors should be readily available for all that can continually advance our noble profession since we still have ground yet to be covered.

*Congratulations to us all.*

9:52 AM - **Anon 46**: Well said.

Thanks Sir🙏🌹

6:48 AM - **Anon 17**: <Media omitted>

Be our guest once again as we learn from Dr. Sheela Ramachandran. *See you in class*. Link is: https://meet.google.com/sue-ykka-ewq

12:37 PM - **ANON 1**: *ATTENTION*!

This is a very important announcement:

All activities of state chapters, councils, directorates and specialty groups requiring graphic design should pass through the PR-CREW for inputs before they're eventually disseminated. This is to ensure uniformity and maintain the standard of ACAPN designs as far as publicity is concerned.

When coming up with a design, the following two factors amongst others must be in place:

* The originality of the ACAPN logo much be maintained. It shouldn't be altered by any means.

* There must be a blend of ACAPN colour (the colour of our T-shirt) with the shade of green which our logo has. A blend of that shade of yellow and the shade of green (lemon) which our logo has will be okay.

Finally, all Councils, chapters and specialty groups with intending educational activities should send it to the scientific council for approval before they embark on it. In the same vein, all non-educational activities should be sent to the NEC for approval.

Thank you.

God bless ACAPN!

8:36 PM - **Anon 33**: 🤝

9:05 PM - **Anon 75**: Thank you our PRO

12:11 PM - **Anon 4**: Hello Senior Colleagues and Colleagues, I hope you are all keeping safe and healthy.

God bless you and all yours.

We will all excel in all spheres.

Sincerely,

❤

Dr. **P5**

12:15 PM - **Anon 33**: STK-20200401-WA0036.webp (file attached)

1:37 PM - **Anon 18**: STK-20210221-WA0035.webp (file attached)

1:37 PM - **Anon 18**: STK-20210221-WA0035.webp (file attached)

1:45 PM - **Anon 41**: STK-20210208-WA0039.webp (file attached)

1:45 PM - **Anon 15**: STK-20201224-WA0034.webp (file attached)

1:45 PM - **Anon 41**: STK-20210221-WA0036.webp (file attached)

11:57 PM - **ANON 1**: <Media omitted>

The Association of Clinical and Academic Physiotherapists of Nigeria (ACAPN) holds Ordinary General Meeting (OGM) on March 13, 2021!

Agenda include *Bye-Election for the office of the Xxxxxx, General Sccc* and Any other Business.

*Qualification*:

In line with Article 36.2 of ACAPN Constitution, the *xxxxxxial nominee* shall have graduated from any MRTB accredited Physiotherapy programme for a period of *not less than twelve (12) years. For the post of the General Sccc, he/she must have graduated for not less than a period of ten (10) years according to Article 36.3 of ACAPN Constitution*.

In the same vein, such individuals must have registered with the association as a full member and *paid ACAPN dues up-to-date without any outstanding dues/levies* according to Article 36.1 of ACAPN Constitution.

To get the nomination form for the election, click on the link below:

https://s.docworkspace.com/d/AIsqNGXt-stbiLmF46adFA

Long live ACAPN!

Long live Physiotherapy Profession in Nigeria!

11:59 PM - **ANON 1**: *Note that the nomination form is both for the office of the Xxxxxx and that of the General Sccc*!

4:29 AM - **Anon 49**: https://mymentalhealthrisk.creighton.edu

8:56 AM - **Anon 14** added **Anon 95**

10:36 AM - **Anon 58**: This message was deleted

10:37 AM - **Anon 58**: This message was deleted

10:37 AM - **Anon 58**: **Anon 109** welcome on board of progressive PTs.

10:56 AM - **Anon 58**: https://www.gov.uk/government/news/government-launches-health-and-care-visa-to-ensure-uk-health-and-care-services-have-access-to-the-best-global-talent

10:58 AM - **Anon 58**: Dm **Contact 16**for details/assistance? Cheers.

11:37 AM - **Anon 58**: Pls feel free to chat with on whatsapp

3:58 PM - **Anon 55**: DPT Program approved by the University of Xxxxxxx Senate.

Congratulations!

3:59 PM - **Anon 7**: Great! Good news.

4:00 PM - **Anon 33**: Doctor of Physiotherapy (DPT) Programme approved by the University of Xxxxxxx Senate today, Wednesday February 24th 2021.

Congratulations to the Physiotherapy family in Nigeria.

4:01 PM - **ANON 1**: Great!

4:01 PM - **ANON 1**: Great👏👏

4:09 PM - **Anon 7**: Thank God.

Kudos to the academic Physiotherapists of UNILAG extraction, MRTB, Physiotherapy clinicians and others that were tools to the Senate approval.

The transition of current B.Sc to DPT should equally be worked out to make it gain ground faster.

4:25 PM - **Anon 57**: I want to sincerely appreciate the entire faculty of the department of Physiotherapy CMUL and in particular our esteemed professors and senior members of faculty who spent several hours drafting and redrafing the curriculum before it was deemed acceptable by the University. God bless everyone and the Physiotherapy family in Nigeria

5:12 PM - **Anon 76**: Hearty cheers to them

6:17 PM - **Anon 38**: Heartwarming news indeed. Congratulations!

6:27 PM - **Anon 46**: Great news.

Congratulations Physiotherapy Dept, Unilag.

3:29 AM - **Anon 5**: This is great! 👏🏾👏🏾👏🏾👏🏾🤝🏾

5:31 AM - **Anon 73**: I made payment of my annual membership registration fee and yet to be acknowledged. **P53** **P17** **P17**. Good morning

9:41 AM - **Anon 9**: STK-20210301-WA0018.webp (file attached)

9:41 AM - **Anon 9**: STK-20210301-WA0018.webp (file attached)

10:54 PM - **Anon 55**: This message was deleted

10:34 AM - **Anon 15**: Annual dues for **P47** received and acknowledged with thanks. So sorry it came late sir

11:45 AM - **Anon 73**: Thank you very much

12:13 PM - **Anon 15**: 🙏🙏🙏

1:29 PM - **Anon 73**: <Media omitted>

7:38 PM - **ANON 1**: <Media omitted>

The Ordinary General Meeting (OGM) of ACAPN now holds on *March 27, 2021*.

Agenda include *Bye-Election for the office of the Xxxxxx, General Sccc* and Any other Business.

*Qualification*:

In line with Article 36.2 of ACAPN Constitution, the *xxxxxxial nominee* shall have graduated from any MRTB accredited Physiotherapy programme for a period of *not less than twelve (12) years. For the post of the General Sccc, he/she must have graduated for not less than a period of ten (10) years according to Article 36.3 of ACAPN Constitution*.

In the same vein, such individuals must have registered with the association as a full member and *paid ACAPN dues up-to-date without any outstanding dues/levies* according to Article 36.1 of ACAPN Constitution.

All completed nomination forms must be sent to the electoral committee xxxxxxx on **P54**. To get the nomination form, click on the link below:

https://s.docworkspace.com/d/AIsqNGXt-stbiLmF46adFA

Long live ACAPN!

2:20 PM - **Anon 13**: Topic: Ogun ACAPN Webinar Series

Time: Mar 4, 2021 03:00 PM West Central Africa

Join Zoom Meeting

https://ukzn.zoom.us/j/95500059165?pwd=OHpUNURibmdiSlorM29IRFlwam5vQT09

Meeting ID: 955 0005 9165

Passcode: 959695

2:20 PM - **Anon 13**: <Media omitted>

2:45 AM - **Anon 77**: *A thousand and one questions Ghanaians ask their government as vaccine arrives*🤔🤔🤔

Here are 12 important questions and answers before considering getting vaccinated:

●"If I get vaccinated can I stop wearing a mask(s)?"

Goverment: "NO"

●"If I get vaccinated will the restaurants, bars, schools, fitness clubs, hair salons, etc. reopen and will people be able to get back to work like normal?

Government: "NO"

●"If I get vaccinated will I be resistant to Covid?"

Government: "Maybe. We don't know exactly, but probably not."

●"If I get vaccinated, at least I won't be contagious to others - right?"

Government: "NO. the vaccine doesn’t stop transmission."

●"If I get vaccinated, how long will the vaccine last?"

Government: "No one knows. All Covid "vaccines" are still in the experimental stage."

● "If I get vaccinated, can I stop social distancing?"

Government: "NO"

● "If my parents, grandparents and myself all get vaccinated can we hug each other again?"

Government: "NO"

● "So what's the benefit of getting vaccinated?"

Government: "Hoping that the virus won't kill you."

●"Are you sure the vaccine won't injure or kill me?"

Government: "NO"

●"If statistically the virus won't kill me (99.7% survival rate), why should I get vaccinated?"

Government: "To protect others."

●"So if I get vaccinated, I can protect 100% of people I come in contact with?"

Government: "NO"

● "If I experience a severe adverse reaction, long term effects (still unknown) or die from the vaccine will I (or my family) be compensated from the vaccine manufacture or the Government?"

Government: "NO - the government and vaccine manufactures have 100% zero liability regarding this experimental drug"

So to summarize, the Covid19 "vaccine"...

Does not provide immunity

Does not eliminate the virus

Does not prevent death

Does not guarantee you won’t get it

Does not stop you from passing it on to others

Does not eliminate the need for travel bans

Does not eliminate the need for business closures

Does not eliminate the need for lockdowns

Does not eliminate the need for masking

Question:

So what does the vaccine do?

Can Nigerians ask the same questions before taking vaccines ?

I rest my case.

10:47 AM - **Anon 15**: 2021 annual dues for **P83** received and acknowledged with thanks

10:49 AM - **ANON 1**: *Call for submission of Articles*!

This is a reminder!

We hereby call for submission of articles for the *3rd edition of our Absolute Physiotherapy Magazine*.

Absolute Physiotherapy Magazine is the official Magazine of the Association of Clinical and Academic Physiotherapists of Nigeria (ACAPN). We want to receive good articles from members of ACAPN on different areas. For example, one of our outline is *news* which could either be local, national or international! Of course these will be current/interesting physiotherapy news. We want to have them in our magazine!

Furthermore, we also have another section we call *features*! This area showcases different scientific articles on different areas of Physiotherapy. For example, we had a caption like "challenges of Women's Health and Physiotherapy education: clinical and academic perspective" in the last edition.

Therefore, the magazine is not only the affairs of the PR-CREW members alone. It's an ACAPN affair! That's why we are calling for articles from our members who are able to make their own contributions as well. It will make the magazine better!

In order to get the magazine produced on time, we are giving from now to the end of *March 2021* for us to submit our articles. This extension is for members to still have the opportunity of participating. All articles should be submitted to the ACAPN PRO via email (**Email 1**).

Thank you as you join to make ACAPN better and also rebrand the Physiotherapy profession!

Long live ACAPN!

PR-CREW, ACAPN.

10:57 AM - **Anon 6**: 👍👍

11:02 AM - **ANON 1**: <Media omitted>

The Ordinary General Meeting (OGM) of ACAPN now holds on *March 27, 2021*.

Agenda include *Bye-Election for the office of the Xxxxxx, General Sccc* and Any other Business.

*Qualification*:

In line with Article 36.2 of ACAPN Constitution, the *xxxxxxial nominee* shall have graduated from any MRTB accredited Physiotherapy programme for a period of *not less than twelve (12) years. For the post of the General Sccc, he/she must have graduated for not less than a period of ten (10) years according to Article 36.3 of ACAPN Constitution*.

In the same vein, such individuals must have registered with the association as a full member and *paid ACAPN dues up-to-date without any outstanding dues/levies* according to Article 36.1 of ACAPN Constitution.

All completed nomination forms must be sent to the electoral committee xxxxxxx on **P54**. To get the nomination form, click on the link below:

https://s.docworkspace.com/d/AIsqNGXt-stbiLmF46adFA

Long live ACAPN!

3/5/21, 11:06 AM - **ANON 1**: Concerning the election, *the system is online voting and there will be password for financial members*!

This means that only financial members will have a password to the platform.

Ensure that you are up to date with your dues!

Long live ACAPN!

1:46 PM - **Anon 13**: This message was deleted

1:49 PM - **Anon 78**: What time will the meeting take place

2:01 PM - **Anon 13**: <Media omitted>

2:02 PM - **Anon 13**: Thanks for the obx

2:07 PM - **Anon 78**: You are welcome

2:08 PM - **Anon 6**: 👏👏👏

2:08 PM - **Anon 17**: Well done Ogun state chapter.

We are proud of you

2:09 PM - **Anon 13**: 👍🏿👌🏿👏🏿

5:33 PM - **Anon 15**: 2021 annual dues for **Anon 4** received and acknowledged with thanks

1:02 PM - **Anon 15**: <Media omitted>

Please send a message to my inbox when your annual dues payment is made. Xxxxxx ACAPN

2:30 PM - **Contact 7**: As professionals and committed members of ACAPN, I will encourage you to pay up your annual dues in order for us to execute programmes for our collective professional development. Remember:

1. Our dues are cheaper

2. We have better development programme

3. We have more educative programme

4. We have world class mentors

5. We have more resources

6. We have integrity

7. We are fully professional and ethical

And many more

WE ARE ACAPN, the CNS and prime movers of Physiotherapy Profession

2:42 PM - **ANON 1**: <Media omitted>

Have you paid your dues? If no, try and do so! Please send a message to ACAPN xxxxxx on **Contact 10** when your annual dues payment is made. Thank you!

2:44 PM - **ANON 1**: <Media omitted>

Have you paid your dues? If no, try and do so! Please send a message to ACAPN xxxxxx on **Contact 10** when your annual dues payment is made. Thank you!

3:38 PM - **Anon 15**: 2021 annual dues for **P13** received and acknowledged with thanks

9:48 AM - **Anon 15**: 2021 annual dues for **P57** received and acknowledged with thanks

12:45 PM - **Anon 15**: 2020/2021 annual dues for **Anon 60** received and acknowledged with thanks

2:51 PM - **Anon 15**: 2020/2021 annual dues for **P58** received and acknowledged with thanks

3:12 PM - **Anon 15**: 2021 annual dues for **Anon 41** received and acknowledged with thanks

5:55 AM - **Anon 17**: IMG-20210310-WA0000.jpg (file attached)

3/8/21, 5:56 PM - **Contact 7**: Happy international women's day. As a gift for today, I am paying the annual due for all the women...

Not less than 80 years of age

6:33 PM - **Anon 4**: STK-20210217-WA0032.webp (file attached)

6:33 PM - **Anon 4**: STK-20210222-WA0019.webp (file attached)

8:10 PM - **ANON 1**: <Media omitted>

The Association of Clinical and Academic Physiotherapists of Nigeria (ACAPN) wishes you Happy International Women's Day celebration!

9:20 PM - **Anon 79**: 👌👌👌🥳🥳🥳💃💃💃

10:01 PM - **Anon 80**: 🙏🏽

10:23 PM - **Anon 33**: <Media omitted>

10:23 PM - **Anon 33**: Then you should be come a Professor of geriatrics

11:36 PM - **ANON 1**: Thank you my brother!

5:17 AM - **Anon 9**: https://l.kphx.net/s?d=286526574409331042

6:20 AM - **Anon 33**: UNILAG School of Postgraduate Studies Announces Dates for 2020/2021 Qual**Anon 106**ing Examination

*Facebook:* ***fb 1***

*Website:* https://unilag.edu.ng/?p=8031

*Twitter:* **twitter 2**

*Instagram:* ***IG 1***

*LinkedIn:* ***Linked 1***

*Google:* https://posts.gle/pmvJS

8:26 AM - **Anon 15**: 2021 annual dues for ***P60*** received and acknowledged with thanks

8:59 AM - **Anon 15**: 2021 annual dues for Dr **P17** received and acknowledged with thanks

7:51 PM - **ANON 1**: *Attention*!

This is a very important announcement!

If you registered for WCPT conference as an NSP member, kindly contact our acting Xxxxxx **P46** on **Contact 7** or the Asst. Sccc, **Anon 14** on **Contact 11**.

This will enable us sort things out immediately.

Thank you!

7:53 PM - **Anon 9**: Which conference???

8:14 PM - **ANON 1**: World Confederation for Physical Therapy (WCPT) conference 2021.

8:26 PM - **ANON 64**: Noted sir

9:07 PM - **Anon 79**: This message was deleted

9:08 PM - **Anon 79**: Good development.👌👌👌

2:09 PM - **ANON 64**: I am looking for a physiotherapist to be based with my football players in Shagamu that is conversant with sports medicine management

Thank you

2:10 PM - **ANON 64**: Please note: Must be Shagamu-based can’t be coming from outside. Prefarably someone that works in a hospital it’s Part time.

2:10 PM - **ANON 64**: Please chat me up if you are interested or you know anyone. Thanks

3:09 PM - **Anon 13**: Topic: OGUN ACAPN WEBINAR SERIES

Time: Mar 11, 2021 03:00 PM West Central Africa

Join Zoom Meeting

https://ukzn.zoom.us/j/95831142437?pwd=N0hlcTBKOFZyMWhNSGwrcU5rbFc1UT09

Meeting ID: 958 3114 2437

Passcode: 291824

3:09 PM - **Anon 13**: <Media omitted>

3:10 PM - **Anon 17**: Una well done👍

*Please let's join in*🙏

3:42 PM - **Anon 33**: STK-20200401-WA0050.webp (file attached)

4:07 PM - **Anon 4**: Tried joining to no avail.

7:06 PM - **Anon 80**: **Contact 12**, **ANON 110** Sir

7:07 PM - **Anon 6**: **P61**🙌🙌🙌

8:41 PM - **Anon 15**: This message was deleted

8:51 PM - **Anon 15**: <Media omitted>

Please send a message with proof of payment when your annual dues are paid. Xxxxxx

8:23 AM - **ANON 64**: Can this person or you dm me with full name and contact please. Thanks🙏

8:23 AM - **ANON 64**: Oga mi I be messenger o🤣🤣🤣

1:50 PM - **Anon 15**: This message was deleted

2:18 PM - **Anon 15**: 2020/2021 annual dues for **Anon 59** received and acknowledged with thanks

2:35 PM - **ANON 1**: <Media omitted>

The Ordinary General Meeting (OGM) of ACAPN now holds on *March 27, 2021*.

Agenda include *Bye-Election for the office of the Xxxxxx, General Sccc* and Any other Business.

*Qualification*:

In line with Article 36.2 of ACAPN Constitution, the *xxxxxxial nominee* shall have graduated from any MRTB accredited Physiotherapy programme for a period of *not less than twelve (12) years. For the post of the General Sccc, he/she must have graduated for not less than a period of ten (10) years according to Article 36.3 of ACAPN Constitution*.

In the same vein, such individuals must have registered with the association as a full member and *paid ACAPN dues up-to-date without any outstanding dues/levies* according to Article 36.1 of ACAPN Constitution.

All completed nomination forms must be sent to the electoral committee xxxxxxx on **P54**. To get the nomination form, click on the link below:

https://s.docworkspace.com/d/AIsqNGXt-stbiLmF46adFA

Long live ACAPN!

2:36 PM - **ANON 1**: Concerning the election, *the system is online voting and there will be password for financial members*!

This means that only financial members will have a password to the platform.

Ensure that you are up to date with your dues!

Long live ACAPN!

6:45 PM - **Anon 15**: 2021 annual dues for **Anon 111** received and acknowledged with thanks

10:22 PM - **ANON 1**: *Attention*!

This is a very important announcement!

If you registered for this year's WPT conference as an NSP member, kindly go ahead and complete your payment to WPT as a non-member on WPT website.

This is based on issues that arose at the previous WPT conference relative to registration fees and other related matters. On this note, the ACAPN NEC advises members who registered for this year's conference to update their registration as non-members. We are sorry for the inconveniences.

Thank you!

10:56 PM - **Anon 41**: So why is ACAPN just saying this now? This should av been made known since call for Abstracts etc was opened for the Conf!

11:12 PM - **Anon 47**: PTT-20210312-WA0068.opus (file attached)

8:44 AM - **Anon 60**: Did ACAPN ever asked anyone to register as NSP members in the first place? Did anyone seek clarification before going on register as NSP member? We were asked to register as NSP members for Geneva 2019 by WPT and those of us that did, later had issues as NSP started their usual dirty politics and WPT did not help matters. Nobody will want to have a repeat of that experience, and I did not see any instruction this year, asking our members to register under NSP. Those that did, cannot blame ACAPN for their deeds.

12:10 PM - **ANON 1**: Highly esteemed colleagues,

The Nigerian Journal of Physiotherapy and Rehabilitation is the official publication of the Association of Clinical and Academic Physiotherapists of Nigeria (ACAPN). It aims at providing an avenue for researchers and practitioners from various specialties in physiotherapy and other rehabilitation-related disciplines to share their ideas and research findings, and foster the use of research in forming public policy.

Are you an author? Do you want to contribute to the journal? Click on the link below to get the details you need:

https://s.docworkspace.com/d/AKBMm8Dt-stbsPvN2qadFA

Long live ACAPN.

Long live Physiotherapy Profession in Nigeria!

12:21 PM - **Anon 73**: In my opinion ACAPN has no blame in this matter. I contacted the organisers of the WCPT conference and I was informed that I can only register as a non member if I am not a member of NSP and certainly I am not a member of NSP. This only brings up the issue of ACAPN working towards being recognised and accepted by WCPT

12:47 PM - **Anon 59**: ✔️ ACAPN is working on that. God's willingly during this year's AGM all information will be furnished to the entire house please everyone let's make plans to attend this year conference

12:49 PM - **Contact 7**: Good morning Aunty. I do understand with you on the question ma. I think people should have clar**Anon 106** with the NEC before going ahead to register especially when they did not find ACAPN on the list.

1:09 PM - **Contact 7**: My Oga sir, you're very right sir. We actually applied for membership with World Physiotherapy before Geneva. That's why we're officially invited to the General Meeting that held before the Geneva conference. At the GM, there's debate for registration of two national associations per country. This scaled through at the first and second readings and overwhelmingly voted for. However, it was brought again for debate after the break and was not approved. At the reconciliatory meeting with the World Physiotherapy CEO and secretariat, after every effort for reconciliation didn't scale through, it's said that the onè with higher members will be recognised. Then thè game started. Despite it's obviously clear that ACAPN had higher members, they started inflating their numbers which we cannot do for our integrity. We decided to stay put and watch the events. Unfortunately for them, the failed to realise that you pay to World Physiotherapy per number o members declared. We know they will hit th rock as soon as payment is asked for. This prompt the present action. I know the NEC will do the needfull soon concerning registration with World Physiotherapy.

Thank you sir

9:43 PM - **Anon 5**: <Media omitted>

9:44 PM - **Anon 5**: Thanks for the clarification sir 🤝🏾

10:19 AM - **Anon 3**: 👍🏽

2:02 PM - **Anon 81**: STK-20210314-WA0027.webp (file attached)

5:19 AM - **Anon 25**: https://pharmanewsonline.com/nuc-upgrades-bachelor-of-physiotherapy-programme-to-doctor-of-physiotherapy/?amp

6:49 AM - **ANON 1**: *Attention*!

This is a very important announcement:

If you registered for this year's WPT conference as an NSP member and want to complete your registration as a non-member as earlier directed, kindly forward your name to our acting Xxxxxx, **P46**, on **Contact 7** for compilation. They will thereafter be forwarded collectively.

This issue is best handled collectively for best outcomes. Let us note that NSP can only ident **Anon 106** her financial members to confirm their registration with WPT. Thus, ACAPN members will not be identified by NSP in order to confirm their registration.

We, the NEC, will continue to serve you and will never take your confidence in us for granted.

Thank you.

7:33 AM -**Anon 20**: NOTICE

Ogun State Hospitals Management Board is recieving applications from interested qualified Individuals for the following Positions:

1. Consultant General surgeon

2. Consultant Ophthalmologist

3. Medical officer

4 Dental officer

5. Pharmacist

6. Physiotherapist

7. Nursing Officer (including Dental Nurses)

8. Medical Laboratory Scientist

9. Optometrist

10.Biomedical Engineer

11. Medical Records Officer

12. Radiographer/Image scientist

13.Dental Technologist

14. Dental Therapist

15.Health Technician (Dental Surgery)

16.Medical Records Technician

17. Pharmacy Technician

18. Medical Laboratory Technician

19. Prosthetics & Orthotic Technician

Submission of Applications addressed to the Permanent Sccc, Hospitals Management Board, Block A Room 149 , New Secretariat Oke mosan, Abeokuta opens on Monday 15th March 2021 and closes Friday 26th March 2021. Only Shortlisted candidates will be contacted for the oral interview.

E-Signed

Management

11:22 AM - **Anon 59**: https://youtu.be/sGL0hckWeVo

**Anon 4** fondly called mummy mi by me has yet again made us by proud by this beautiful presentation delivered to Prentice Institute.

Please enjoy and happy viewing

11:24 AM - **Anon 6**: 👏👏👏👏

11:50 AM - **Anon 59**: ✔️✔️❤️

12:36 PM - **Anon 46**: 🌹🙏

5:21 PM - **Anon 61**: 👍👍👍

5:23 PM - **ANON 1**: Well done Ma👏👏

11:52 PM - **Anon 75**: STK-20210301-WA0014.webp (file attached)

11:52 PM - **Anon 75**: CONGRATULATIONS ma

3:31 AM - **Anon 41**: Congratulations Ma. 👏👏👏👏

5:41 AM - **Anon 82**: 👏🏼👏🏼👏🏼

11:53 AM - **Anon 59**: ‼️‼️‼️‼️‼️‼️‼️‼️‼️‼️‼️‼️‼️‼️‼️‼️

📌 Do you love books ? *📚📚* 📌Are you an aspiring to be an author but have no clue on how to go about publishing it?* 😥

🖊️🖊️🖊️🖊️🖊️🖊️🖊️

📌 *Do you desire to put your thoughts into words but have neither had the opportunity nor courage to do so?

💥💥💥💃🏻💃🏻💃🏻

Here's your chance to actualize your dreams and receive a 3 day free training on a platter of gold!!!

In addition you will earn cool cash.

💰💰💰

Why miss this rare opportunity?

Join our team for a super training on how to write and self- publish your ebooks without stress.

🕺🕺🕺🕺🕺🕺🕺🕺

🏃🏻‍♀️🏃🏻‍♀️🏃🏻‍♀️🏃🏻‍♂️🏃🏻‍♂️🏃🏻‍♂️🏃🏻‍♂️Rush NOW and click the link here 👇 to join the

WRITE- FOR - ME

TRAINING*** Free

https://chat.whatsapp.com/DQA1RY5zpuJKKXBW6EQXzr

11:55 AM - **Anon 3**: 😁You make us proud ma🙏🏽💪🏽

12:02 PM - **Anon 49**: **Anon 4**, Whaoooo..Kare o jare. You are doing us proud. God bless you . 👏👏👏👏👏👏🙏🙏🙏🙏

12:23 PM - **Anon 57**: Congratulations my dear sister 👏👏👏👏. Welldone. Greater heights still in Jesus name.

2:39 PM - **Anon 78**: Congratulations and well done ma

2:40 PM - **Anon 83**: Congratulations Ma.

4:31 PM - **Anon 4**: Thank you **P64**. I appreciate

4:31 PM - **Anon 4**: STK-20210217-WA0032.webp (file attached)

4:31 PM - **Anon 4**: STK-20210217-WA0032.webp (file attached)

4:32 PM - **Anon 4**: STK-20210317-WA0026.webp (file attached)

4:32 PM - **Anon 4**: STK-20210317-WA0028.webp (file attached)

4:32 PM - **Anon 4**: STK-20210317-WA0027.webp (file attached)

4:32 PM - **Anon 4**: STK-20210217-WA0032.webp (file attached)

4:33 PM - **Anon 4**: STK-20210317-WA0028.webp (file attached)

4:33 PM - **Anon 4**: STK-20210317-WA0029.webp (file attached)

4:33 PM - **Anon 4**: Thank you **P1**

4:34 PM - **Anon 4**: Thank you sir. Mo dupe sir

4:34 PM - **Anon 4**: Thank you ma

4:34 PM - **Anon 4**: STK-20210317-WA0026.webp (file attached)

4:34 PM - **Anon 4**: STK-20210217-WA0032.webp (file attached)

4:40 PM - **Anon 61**: Congratulations and welldone ma

4:49 PM - **Anon 4**: STK-20210317-WA0027.webp (file attached)

4:58 PM - **Anon 58**: This message was deleted

4:59 PM - **Anon 58**: STK-20210301-WA0014.webp (file attached)

5:03 PM - **Anon 15**: *2021 annual dues for the following PT's from ogun state received and acknowledged with thanks*

**P65**

**P66**

**P67**

**068**

**P69**

**P70**

**P71**

**P72**

**P73**

**P74**

**P75**

**P76**

Apologies if the titles are not all correct, thanks and God bless you all.

6:24 PM - **Anon 6**: 👏👏👏

6:42 PM - **Anon 15**: 2021 annual dues for **P78** received and acknowledged with thanks

6:55 PM - **Anon 59**: Mummy mi ❤️❤️

6:55 PM - **Anon 83**: STK-20201024-WA0072.webp (file attached)

6:55 PM - **Anon 83**: STK-20210317-WA0034.webp (file attached)

6:55 PM - **Anon 83**: STK-20210317-WA0033.webp (file attached)

6:55 PM - **Anon 83**: STK-20210317-WA0032.webp (file attached)

7:05 PM - **Anon 15**: 2021 annual dues for **Anon 57** received and acknowledged with thanks

7:30 PM - **Anon 42**: https://twitter.com/RSUTH_PH/status/1372209722158354443?s=08

7:41 PM - **Anon 4**: Thank you ma

8:09 PM - **Anon 58**: Wow! This is laudable. Well done ogun members.

8:11 PM - **Anon 59**: ✔️✔️✔️👏🏻👏🏻👏🏻👏🏻👏🏻👏🏻

8:12 PM - **Anon 72**: Congratulations **Anon 4**, more upliftment in Jesus name.

8:56 PM - **Anon 7**: Well done ma.

Higher! Higher!! Higher!!!

1:39 AM - **Anon 84** changed to **Contact 13**

1:39 AM - **Anon 84** changed to **Contact 13**

12:02 AM - **Anon 4**: <Media omitted>

AC21 Virtual Flyer (5).pdf

12:02 AM - **Anon 4**: <Media omitted>

SSPN

12:02 AM - **Anon 4**: <Media omitted>

1:12 AM - **Anon 59**: https://rdcu.be/cgSHE

27th Annual Conference of the International Society for Quality of Life Research. Qual Life Res 29, 1–196 (2020). https://doi.org/10.1007/s11136-020-02626-y.

You may want to view our articles 3070 and 3148, other interesting abstract for your consumption.

Happy reading

1:12 AM - **Anon 59**: <Media omitted>

2020_Article_27thAnnualConferenceOfTheInter-1.pdf

1:36 PM - **Anon 13**: Join Zoom Meeting

https://ukzn.zoom.us/j/95775621708?pwd=NFNNZStMblFBUkY0RmxMMU5DSUt0QT09

Meeting ID: 957 7562 1708

Passcode: 495054

1:36 PM - **Anon 13**: <Media omitted>

1:36 PM - **Anon 13**: Reminder

1:36 PM - **Anon 13**: 3pm to 4pm today

1:36 PM - **Anon 13**: Pls make it a date

10:34 AM -**Anon 20**: **P77**, Senior Lecturer in the Department of Medical Rehabilitation, Obafemi Awolowo University (OAU), Ile-Ife, Nigeria, has been conferred a “Visiting Research Fellow” by the Manchester Metropolitan University (MMU), United Kingdom. By this appointment, he will work with **P78** (Professor of Health Economics and Outcomes), **P79** (Professor of Physiotherapy) and colleagues within the Department of Health Professions between 1st March 2021 and 29th February 2024 in the first instance to strengthen and advance collaborations between MMU and OAU in research and learning exchange, as well as other associated benefits for both institutions.

Please celebrate him with me.

Dean – Faculty of Basic Medical Sciences, OAU.

10:36 AM - **Anon 50**: Hearty Congratulations sir 🎉🎉🍾🍾

Your passion and drive keeps paying up and You keep making much more strides into greatness in Jesus name Amen.

Congratulations again sir **Contact 14**

10:37 AM - **Anon 85**: Congratulations sir

10:38 AM - **ANON 86**: Congratulations sir , more of it to come

10:39 AM - **Anon 87**: Congratulations Chief. It's well deserved sir.

You have always been a great mentor when it comes to research and Physiotherapy practices as a whole.

Higher and higher you, Chief👏👏👏👏 **Contact 14**

10:39 AM - **Anon 49**: Congratulations...!

Congratulations....!!

Congratulations **P77**. ..!!!

👏👏👏👏👏👏🙏🙏🙏

10:44 AM - **Anon 17**: Congratulations Sir.

Very very well deserved

10:49 AM - **Anon 7**: Congratulations to him.

10:51 AM - **Anon 6**: Good news.

Congratulations to **P77**

11:07 AM - **Anon 5**: Wao! 👏🏾👏🏾👏🏾👏🏾👏🏾

Congratulations @**P77** 🤝🏾🤝🏾

11:12 AM - **Anon 58**: Wow! Congratulations to you, **P77**. Higher heights, and greater glory for you

11:13 AM - **Anon 3**: 👍🏽nice one sir **Contact 14**

11:13 AM - **Anon 15**: Congratulations to you sir

11:14 AM - **Anon 13**: Congratulations to **P77**

11:21 AM - **Anon 40**: Congratulations **P77**. Higher heights.

11:24 AM - **Contact 2**: Congratulations sir..more to be heard

Greater height sir

11:32 AM - **Anon 59**: Wow congratulations **P77**, your tenacity with research has been nothing but overwhelmingly inspiring. Well done sir

11:42 AM - **Anon 73**: Big congratulations to you **P77**

11:48 AM - **Anon 88**: This message was deleted

11:52 AM - **Anon 33**: STK-20210319-WA0004.webp (file attached)

11:52 AM - **Anon 33**: A gold fish has no hiding place.

Ride on Doc.

More comimg soonest

11:56 AM - **Anon 55**: Awwwn

Congratulations Sir

Ride on

12:05 PM - **Anon 58**: Congratulations Sir

12:05 PM - **Anon 42**: We congratulate our erudite academic n bow before Baba God for making this possible.

12:23 PM - **Anon 46**: Congratulations Sir.

Greater height🌹🙏

12:38 PM - **Anon 34**: Congratulations to you **P77**.

12:38 PM - **ANON 1**: Super congratulations to you Sir.

Greater heights ahead!

Cheers!

12:45 PM - **Anon 89**: A wonderful milestone. Keep firing all cylinders oga mi.

12:48 PM - **Anon 4**: Congrats **P77**. Greater heights. Congrats to the Department of Medical Rehabilitation, OAU. 👏🏾👏🏾🕺🏾🥁🥁

1:00 PM - **Anon 41**: STK-20200906-WA0025.webp (file attached)

1:00 PM - **Anon 41**: Big Congratulations **P77**. So proud of u!

1:20 PM - **Anon 72**: Congratulations **P77**,up up you shall go in Jesus name

1:22 PM - **Anon 76**: A big congrats to you Chief

1:32 PM - **Anon 38**: Congratulations to you **P77**.

1:37 PM - **Anon 90**: Congratulations sir! More wins💃🏻💃🏻💃🏻

1:47 PM - **Anon 54**: Congratulations my Oga @**P77**.

2:29 PM - **Anon 61**: 👍👍👍congratulations sir

3:40 PM - **Anon 83**: Congratulations Sir

4:04 PM - **Anon 90**: Wahoo, good news. **P77**, congratulations👍👍 👏👏🕺🕺🍾🥂🍹

4:39 PM - **Anon 44**: Congratulations **P77**. We are really proud of you. Congrats to OAU Medical Rehabilitation Department too. Greater prospects for the department

4:46 PM - **Anon 91**: Congratulations sir, more greater heights

5:36 PM - **Anon 41**: Hearty congratulations to you Dr. **P77.** **P77**.

7:16 PM - **Anon 92**: Congratulations **P77**.

7:16 PM - **Anon 60**: Please can **P77** mentor me?? 😭😭

I need improve ooo! 😭

Congrats sir!

7:22 PM - **Anon 93**: **P77**, congratulations, more of it to come.

7:35 PM - **Anon 70**: Congratulations to **P77**. Another feather to your cap!

9:28 PM - **Anon 36**: Congratulations to my **P77**. God bless you and your family with more honours in Jesus name. Amin!

9:51 PM - **Anon 94**: Congratulations sir

10:15 PM - **Anon 62**: Congratulations to **P77**.

3/20/21, 9:23 AM - **P77**: Dear Sir/Ma,

I thank you immensely for your messages and good wishes. May God bless you real good. Amen

3/20/21, 10:08 AM - **Anon 46**: Amen Sir.

Congratulations once again. Cheers🍹🍾

3/20/21, 8:03 PM - **Anon 26**: Wow, Congratulations to you Prof and congratulations to Dr **P77**. Ride on to greater heights in Jesus mighty name.

3/22/21, 6:14 PM - **Anon 30**: A big congratulation. The Lord will perfect His purpose upon your life IJMN

3/22/21, 6:44 PM - **Anon 15**: 2021 annual dues for ACAPN Osun state health management board received and acknowledged with thanks.

1. **P80**
2. **P81**
3. **P82**
4. **P83**
5. **P84**
6. **P85**
7. **P86**
8. **P87**
9. **P88**

God bless you all

3/22/21, 7:13 PM - **Anon 89**: Kindly take note that it is "Osun State HOSPITALS' Management Board" and not "Osun State HEALTH Management Board" as indicated here.

7:17 PM - **Anon 89**: STK-20200624-WA0000.webp (file attached)

7:54 PM - **Anon 9**: Aameen

1:16 AM - **Anon 75**: Nice one! The way to go!

6:05 AM - **Anon 33**: Very impressive.

Good commitment.

Following the way of OGUNACAPN.

Winners loading 👍👍👍

10:45 AM - **ANON 1**: *Physiotherapy Wins Again!*

Dear colleagues,

**P89** and **P90** have been re-elected into the Senate of the University of Xxxxxxx. This happened yesterday. Both emerged in the top 20 highest voted among the 150 contestants university-wide 👏👏💃🏻💃🏻.

Let us congratulate them, the CMUL Physiotherapy, the Association of Clinical and Academic Physiotherapists of Nigeria (ACAPN) and the entire Physiotherapy family.

Congratulations to you Sirs. Greater heights ahead!❤️👊

Long live Physiotherapy profession in Nigeria!

10:46 AM - **Anon 78**: Congratulations sirs

10:47 AM - **Anon 95**: Congratulation sirs

10:53 AM - **Anon 6**: Good news.

Congratulations to the ogas.

10:55 AM - **ANON 64**: 👏👏👏👏

11:03 AM - **Anon 5**: Big congratulations my big Ogas 👏🏾👏🏾👏🏾👏🏾👏🏾

Keep the flag flying 🤝🏾

11:22 AM - **Anon 66**: 👏👏👏

11:24 AM - **Anon 62**: Congratulations.

11:25 AM - **Anon 21**: Congratulations dear brothers.

12:06 PM - **Anon 96**: 👍🏾

12:20 PM - **Anon 7**: Congratulations to them, Physiotherapy Dept of CMUL, ACAPN family.

Greater heights.

12:27 PM - **Anon 26**: Congratulations to the duo. Greater heights in Jesus mighty name

12:44 PM - **Anon 3**: Nice one, congratulations to my Ogas

2:31 PM - **ANON 1**: *Updated Timetable for ACAPN Bye Election 2021*

✔️ Vetting of Candidates_ 23/03/2021 4pm.

✔️ Publication of successful candidates list_ 23/03/2021 8pm.

✔️ Campaign_ 24/03/2021 6am. to 26/03/2021 6pm.

All campaigns should be issue based and within Physiotherapy professional scope, policy, administration, politics, and academic and clinical practices.

Candidates discussing with hate speeches, ethnic and religion sentiments may be disqualified.

✔️ Manifesto_ 26/03/2021 6pm to 7pm.

ACAPN Zoom or Google Meet. _Link will be given to financial members._

_Agenda_

1. Opening prayer (5min)

2. Address by Xxxxxxx Electoral Committee (10min)

3. Manifesto by each candidate (7min each).

4. Closing remarks

✔️ Election

27/03/2021.

ACAPN e-voting platform

*Link and password will be sent to financial members.*

Please contact us to know more:

✒️

***P91***

Sccc | Electoral Committee

**Contact 15**

3:51 PM - **Anon 6**: 👏👏👏👏

7:47 PM - **Anon 36**: Congratulations to them.

8:01 PM - **ANON 1**: *ACAPN 2021 Bye Election*

Update | 23/03/2021 | 8pm

*RESULT OF VETTING*

1. **P90** for Xxxxxx ..... *Qualified*

2. **P9** for Sccc ..... *Qualified*

Please contact us to know more:

✒️

***P91**

Sccc | Electoral Committee

**Contact 15**

7:04 AM - **Anon 34**: <Media omitted>

7:44 AM - **Anon 6**: 👍👍👍

7:58 AM - **Anon 65**: STK-20210324-WA0008.webp (file attached)

8:04 AM - **Anon 58**: STK-20210301-WA0014.webp (file attached)

8:05 AM - **Anon 17**: Well done ma'am.👍

8:07 AM - **Anon 49**: Great...!!!!!!!

My salute. Well done. 👏👏👏👏👏🙏🙏🙏🙏👏👏

9:57 AM - **Anon 88**: STK-20210324-WA0013.webp (file attached)

11:34 AM - **Anon 7**: This is beautiful, anxiously waiting.

11:42 AM - **ANON 1**: 👏👏👏👏

4:59 PM - **ANON 29**: Congratulations to all of us 🎉🎉🎉

Point 1 benefits of Council and the Bill passed

Number 1 there will be a regulatory and a statutory body to regulate and pass on the the rules and regulations to the academic as well as clinical criteria practice mechanism

Number 2 you now onwards you will require a licence and registration number to practice the Physiotherapy profession that makes you equally legitimate and curb the false practices

Number 3 improvement in vacancies in private as well as the government sectors

Number 4 licensing towards the academic profession by incorporation of rules and regulation for the intake of seats as well as for the standards required for setting up Physiotherapy College independently or in an University

Number 5 basic requirement as for education will be e The Bachelorette degree that is BPT in government sectors which will raise our standards of practice and grade pay from 4200 towards 4600 and above as per amendments

Number 6 rights to practice independently diagnose investigate and examine in the cases related to the ailment under the scope of practice is allowed which is very clearly mentioned in the Physiotherapy definition under the bill

Number 7 physiotherapist from here on will not be considered under paramedics allied healthcare but under healthcare professionals as a separate stream which shall not be confused here on

Number 8

To raise educational sub standards of learning by checking on regular mode of learning and and putting a halt over distance education in the field of of medical sciences as education is only the matter towards professionalism

4:59 PM - **ANON 29**: <Media omitted>

6:52 PM - **Anon 60**: I did not reveive any link for the campaign this evening oo.

7:09 PM - **Anon 55**: Thank you for asking.

Campaign is the responsibility of individual contestants.

Manifesto date, time and event program is in this timetable.👆🏾👆🏾👆🏾

NEC has subscribed the ACAPN Zoom platform. They will give us the link tomorrow.

You may receive the link and password inbox as a financially up-to-date member.

7:27 PM - **ANON 1**: 👏👏👏

8:40 AM - **Anon 13**: <Media omitted>

8:40 AM - **Anon 13**: OGUN ACAPN WEBINAR SERIES VI

Time: Mar 25, 2021 03:00 PM West Central Africa

Join Zoom Meeting

https://ukzn.zoom.us/j/92978932966?pwd=Q0lmcXNNbjdOdHR2emJMaDFCYzFRUT09

Meeting ID: 929 7893 2966

Passcode: 432489

10:00 AM - **ANON 1**: 👏👏👏👏👏

10:03 AM - **Anon 75**: Ogun ACAPN you make us proud! Well done.

10:04 AM - **ANON 1**: 👏👏👏

2:52 PM - **Anon 13**: This message was deleted

2:52 PM - **Anon 13**: This message was deleted

3:37 PM - **Anon 15**: 2021 annual dues for **Anon 112** received and acknowledged with thanks

4:05 PM - **Anon 15**: 2021 annual dues for **Anon 113** received and acknowledged with thanks

4:18 PM - **Anon 43**: 🙏🏼🙏🏼

4:18 PM - **Anon 13**: 👍🏿

8:34 AM - **ANON 1**: *REMINDER*!

Please be reminded that our Ordinary General Meeting (OGM) holds tomorrow *27th March, 2021 by *5pm prompt*!

I repeat, *5 pm prompt*!

The link for the meeting will be sent before the time.

Please, be prepared and also ensure you are financially up to date!

Thank you!

10:19 AM - **Anon 13**: Topic: OGUN ACAPN WEBINAR SERIES VII

Time: Apr 1, 2021 03:00 PM West Central Africa

Join Zoom Meeting

https://ukzn.zoom.us/j/95898582273?pwd=MzlOdWhMVnBtKzNuMFRkMVg5Mk10UT09

Meeting ID: 958 9858 2273

Passcode: 565783

10:19 AM - **Anon 13**: <Media omitted>

10:26 AM - **Anon 17**: Una just too much.

Any group that has a good leader flies high you in the sky.

Thanks **P93** and the entire Ogun state team.👍

10:26 AM - **Anon 13**: 👍🏿

12:42 PM - **Anon 28**: STK-20200804-WA0010.webp (file attached)

1:01 PM - **Anon 33**: STK-20201120-WA0015.webp (file attached)

2:28 PM - **Anon 15**: 2021 annual dues for **Anon 38** Mellitus received and acknowledged with thanks

3:33 PM - **ANON 1**: *Updated Timetable for ACAPN Bye Election 2021*

✔️ Campaign stops by 6pm today, March 26, 2021.

✔️ Manifesto_holds between 6pm and 7pm today March 26, 2021.

_Agenda_

1. Opening prayer (5min)

2. Address by Xxxxxxx Electoral Committee (10min)

3. Manifesto by each candidate (7min each).

4. Closing remarks

Here is the link for the manifesto:

https://us02web.zoom.us/j/89537305152?pwd=NWJMSTh1VTBtNWk2ejJwRDVhWnFIZz09

Meeting ID: 895 3730 5152

Passcode: 456492

✔️ Election

Voting time: 5pm to 6pm

Results expected: 7pm

ACAPN e-voting platform

*Link and password will be sent to financial members.*

Please contact us to know more:

✒️

***P91***

Sccc | Electoral Committee

**Contact 15**

5:13 PM - **ANON 1**: <Media omitted>

17225.docx

5:15 PM - **ANON 1**: Colleagues,

This is **P90** Manifesto! He is our xxxxxxial nominee.

Don't forget to join in the meeting by 6pm this evening following the shared link.

God bless ACAPN!

5:24 PM - **ANON 1**: **P90** Manifesto/Profile.👆

Don't be left behind. Join in the meeting by 6pm this evening.

Link for the manifesto has been provided on this platform.

God bless ACAPN!

6:09 PM - **ANON 1**: *Please colleagues, join in the meeting for our manifeso. It is ongoing*

6:22 PM - **Anon 55**: ACAPN NIGERIA is inviting you to a scheduled Zoom meeting.

Topic: ACAPN 2021 BYE ELECTION MANIFESTO

Time: Mar 26, 2021 05:50 PM West Central Africa

Join Zoom Meeting

https://us02web.zoom.us/j/89537305152?pwd=NWJMSTh1VTBtNWk2ejJwRDVhWnFIZz09

Meeting ID: 895 3730 5152

Passcode: 456492

7:38 PM - **ANON 1**: *Thank you!*

We, the NEC, appreciate all our highly esteemed members for participating in today's manifeso presentation. We also thank our contestants for good presentation. It was indeed great!

We equally appreciate the electoral committee led by **Anon 118** for a wonderful job.

Tomorrow is the D-DAY. It's our OGM and election proper by 5pm prompt. Please join us once again as we move ACAPN to greater heights!

Thanks and God bless you!

8:49 PM - **Anon 15**: 2021 annual dues for Dr Nse Odunaya received and acknowledged with thanks

10:22 PM - **Anon 95**: <Media omitted

10:24 PM - **Anon 96**: https://anchor.fm/*******-**Anon 96**/episodes/Physiotherapy-Awareness-esj9gf/physiopod-ep1-5-of-14---physiotherapy-sub-specialties-a4uffe8

Please listen and share extensively.

Kindly also drop suggestions, corrections and advice 🙏🏾🙏🏾

7:12 AM - **Anon 38**: Just listened. Nice one there, **Anon 114**!

7:12 AM - **Anon 96**: Thank you so much sir

7:12 AM - **Anon 96**: Any advice or suggestions sir?

7:17 AM - **Anon 38**: Keep it up! If anything, I will dm you.

2:21 PM - **ANON 1**: Good afternoon colleagues,

Hope you are getting set for our OGM/Bye-election coming up by 5pm this evening.

The zoom details for the meeting are as follows:

ACAPN NIGERIA is inviting you to a scheduled Zoom meeting.

Topic: ACAPN 2021 OGM/BYE-ELECTION

Time: Mar 27, 2021 05:00 PM West Central Africa

Join Zoom Meeting

https://us02web.zoom.us/j/82077833303?pwd=ckRRa2EvdFdPSmU5UmoraDFDNDM0Zz09

Meeting ID: 820 7783 3303

Passcode: 782785

3:51 PM - **Anon 3**: 👍🏽👍🏽

5:09 PM - **ANON 1**: Good evening colleagues,

It is already time for the meeting. Please, join in.

The zoom details for the meeting are as follows:

ACAPN NIGERIA is inviting you to a scheduled Zoom meeting.

Topic: ACAPN 2021 OGM/BYE-ELECTION

Time: Mar 27, 2021 05:00 PM West Central Africa

Join Zoom Meeting

https://us02web.zoom.us/j/82077833303?pwd=ckRRa2EvdFdPSmU5UmoraDFDNDM0Zz09

Meeting ID: 820 7783 3303

Passcode: 782785

5:27 PM - **Anon 7**: I am having problem joining meeting.

5:28 PM - **Anon 13**: Use the meeting id

5:28 PM - **Anon 13**: And password

5:36 PM - **Anon 7**: I did and got connected for few seconds and no more. More effort did not yield result.

7:03 PM - **Anon 39**: Congratulations to **Anon 33** and other elected officials.

7:11 PM - **Anon 4**: Colleagues, please join in 🙏🏽🙏🏽

7:34 PM - **Anon 4**: There are important issues to be discussed

7:35 PM - **Anon 38**: Having issues rejoining the meeting.

I have tried the link; also the code and password all to no avail.

Someone please help.

7:41 PM - **Anon 7**: I have similar experience.

7:59 PM - **Anon 34**: You may need to update or reinstall the app.

8:14 PM - **Anon 55**: <Media omitted>

ACAPN CONSTITUTION - final reviewd copy..pdf

1:22 AM - **ANON 1**: *IT'S A NEW DAWN ONCE AGAIN IN ACAPN!*

The Association of Clinical and Academic Physiotherapists of Nigeria (ACAPN) witnessed a transition of the office of the National Xxxxxx and that of the General Sccc during her Ordinary General Meeting (OGM)/Bye-Election that virtually took place on March 27, 2021.

In the Bye-Election, **P90** from **P46**.

Both officers took oath of office and were dully swon into their respective offices according to the Constitution!

ACAPN keeps making progress and getting better by the day.

Congratulations to you Sirs. We wish you a successful tenure in office 👏👏💃🏻💃🏻

Colleagues, join us to congratulate them.

PR-CREW, ACAPN.

5:59 AM - **Anon 7**: I will be happy if resolution can be made available to those of that couldn't link for ty meeting.

7:03 AM - **Anon 7**: Congratulations.

It will be for the upliftment of Physiotherapy in Nigeria.

7:22 AM - **Anon 49**: Congratulations...Mr Xxxxxx...!

Congratulations.

.Mr General Sccc...!!

Congratulations..Mr substantive Auditor... !!!

The Lord God shall empower you all with the wisdom for these offices

To Madam acting Xxxxxx...I also congratulate you for holding the reign forth till the elections.

To our ACAPN family. I congratulate us all . 🙏🙏🙏

11:29 AM - **Anon 6**: 👏👏👏

12:13 AM - **Anon 58**: STK-20210326-WA0023.webp (file attached)

6:59 AM - **Anon 96**: Congratulations @2348023078247 on your new position sir 👏🎉🎊 may God help you to be execute innovations and transform the profession

6:59 AM - **Anon 96**: Thank you ma

7:36 AM - **Anon 6**: IMG-20210329-WA0005.jpg (file attached)

8:38 AM - **Anon 98**: Congratulations **P90**, the Lord will work with you.

To **P46**, the Lord bless you richly for holding forth, wearing two caps.

Together, we shall all make Physiotherapy great.

Long Live ACAPN!

Long Live Physiotherapy!!

9:14 AM - **Anon 34**: https://youtu.be/uUnL3x9W53w

10:11 AM - **Anon 44**: Congratulations to **P90** (Xxxxxx) and **P15** (Sec Gen) on your election. Thanks **Anon 17** and otter Exco members for holding forth all the while and conducting a successful election. Long live ACAPN.Long live all Nigerian Physiotherapists.

10:46 AM - **Anon 62**: Congratulations.

10:56 AM - **Anon 3**: Congrats Sir,....we look forward to greater things👍🏽

11:32 AM - **Anon 99**: Good day colleagues.

This is a questionnaire on the *Acceptance and experience with COVID vaccine among health care workers*. It takes less than 2 minutes to fill. Kindly help fill this google form.

Thanks in anticipation.

https://docs.google.com/forms/d/e/1FAIpQLSfgwykUV2yFex4aMRPpnWPKhmym2fEiGnIdmQsTflwtu9-pNw/viewform?usp=sf_link

10:46 PM - **Anon 83**: Congratulations sir on your appointment as the Xxxxxx of this highly reputable professional association.

The reward of hard work is more work, therefore, I pray that the almighty God will continually bestow on you the wisdom and other things required to take ACAPN to greater heights.

Congratulations sir!

11:06 PM - **Anon 30**: Great!! A big congratulation. The Lord will empower you with wisdom, knowledge & understanding to successfully govern the affairs of ACAPN IJMN. Receive grace.

5:07 AM - **Anon 52**: Congratulations Sir

6:16 AM - **Anon 100**: Congratulations sir

10:26 AM - **ANON 1**: *ATTENTION*!

This is a very important announcement:

Dr. **P77.** E. **P77** has been appointed as the Xxxxxxx, Scientific Council, ACAPN. His functions start immediately.

Congratulations to you Sir 🤝💃🏻. Greater heights ahead!

God bless ACAPN!

10:48 AM - **Anon 6**: 👍👍👍👍

10:54 AM - **Anon 91**: Waoh, Congratulations Sir

11:06 AM - **Anon 15**: 👏👏👏👏

11:27 AM - **Anon 41**: STK-20200522-WA0017.webp (file attached)

2:34 PM - **Anon 13**: Topic: OGUN ACAPN WEBINAR SERIES VII

Time: Apr 1, 2021 03:00 PM West Central Africa

Join Zoom Meeting

https://ukzn.zoom.us/j/95898582273?pwd=MzlOdWhMVnBtKzNuMFRkMVg5Mk10UT09

Meeting ID: 958 9858 2273

Passcode: 565783

2:34 PM - **Anon 13**: <Media omitted>

4:32 PM - **Anon 13**: Copy the link below to share this recording with viewers:

https://ukzn.zoom.us/rec/share/gZ0QPCt-VsJEYzxF7x96TQoEghukHgcGZ7UNNhhBCLbBNpjveMyFwbA27JhxSg.BKDAutKydjpSFbQT Passcode: YKn&7=K?

4:47 PM - **Anon 13**: This message was deleted

4:54 PM - **Anon 61**: 👏👏👏

7:08 PM - **Anon 59**: 👏🏻👏🏻👏🏻

8:30 PM - **Anon 4**: Congrats **P77** 👏🏾👏🏾👏🏾

1:43 PM - **Anon 26**: Congrats Dr **P77**

2:10 PM - **ANON 1**: <Media omitted>

The Association of Clinical and Academic Physiotherapists of Nigeria (ACAPN) wishes you a happy and fulfilling Easter Celebration!💃🏻❤️.

2:16 PM - **Anon 59**: ❤️❤️

2:16 PM - **Anon 33**: 👍👍

2:18 PM - **Anon 55**: Thank you so much

2:22 PM - **Anon 6**: 👍👍👍

3:49 PM - **Anon 34**: 👊👍👏

3:58 PM - **Anon 15**: 👏👏👏👏

3:59 PM - **Anon 56**: No thanks

4:00 PM - **Anon 7**: Prof, thank you sir.

10:01 PM - **Anon 5**: 🤝🏾🤝🏾

10:02 PM - **ANON 64**: 👏👏👏👏

10:03 PM - **Anon 4**: Well done our PRO

2:28 AM - **Anon 17**: 👍👍

8:42 AM - **Anon 33**: EASTER MESSAGE

Dear Colleagues!

Happy Easter to us all.

As individuals and as members of the large family of ACAPN, typified by Integrity, Dignity and Love, may the reason of this Season cause us all to live up to our Professional callings.

May we continue to stand up as examples of true professionals and thus contribute significantly to the growth of our Association, ACAPN and our profession, Physiotherapy.

Thank you all.

**Anon 33**

Xxxxxx, ACAPN.

9:01 AM - **Anon 15**: 💪💪👏👏

9:07 AM - **Anon 3**: 👍🏽

9:35 AM - **Anon 59**: 👌🏻👌🏻❤️❤️❤️

9:57 AM - **Contact 7**: The period of Easter is for celebration of victories. May you continuously be celebrating victories in your life.

**P97**

2:43 PM - **ANON 1**: Thank you Sir👊👊👏👏

2:44 PM - **Anon 4**: STK-20210317-WA0028.webp (file attached)

2:45 PM - **Anon 4**: STK-20210317-WA0027.webp (file attached)

5:18 PM -**Anon 8**: 🙏

6:05 PM - **Anon 115**: Good day Ma.

I am a Postgraduate student in The University of Ibadan, Faculty of Social Sciences, Sociology Department with a Research topic on *The Contraceptive Use of Billings Ovulation Method among Married women in Ibadan.* Kindly help me fill this questionnaire through this link. Thank you ma

https://forms.gle/aTADFdmyV4Q5AhLb8

6:18 PM - **Anon 115**: It is meant for married women, who are still in d reproductive age, and are living within Ibadan Metropolis

8:25 PM - **Anon 58**: <Media omitted>

2:38 PM - **Anon 33**: MSc ENTRANCE INTERVIEW

Date: Tuesday April 6th Time:10am

Venue: Physiotherapy Dept College of Medicine University of Xxxxxxx (Idi-araba).

Kindly note or inform any prospective candidate you may know to come with original credentials and evidence of publications or Awards.

Thank you.

**P90**

7:33 PM - **ANON 1**: <Media omitted>

The Association of Clinical and Academic Physiotherapists of Nigeria (ACAPN) fecilitates with the entire Nigerian Physiotherapists on the event of marking the World Physical Activity day (April 6, 2021).

Happy celebrations!

7:34 PM - **Anon 6**: 👍👍👍

7:40 PM - **Anon 5**: 🤸🏾‍♂️🤾🏾‍♂️🏌🏾‍♂️🏋🏾‍♂️⛹🏿‍♂️🚴🏾‍♂️⚽💪🏾

7:51 PM - **Anon 33**: STK-20201120-WA0015.webp (file attached)

8:04 PM - **Anon 59**: ✔️✔️

8:17 PM - **Anon 15**: 👏👏👏

9:13 PM - **Anon 18**: This message was deleted

9:13 PM - **Anon 18**: This message was deleted

9:14 PM - **Anon 18**: This message was deleted

9:53 PM - **Anon 46**: 🙏🌹

6:08 PM - **Anon 33**: Dear colleagues, please do me the honour of filling out this questionnaire. My team and I are studying Infection Prevention and Control among Healthcare workers in Xxxxxxx, in the light of COVID 19.

Thanks so much.

**Anon 33**

**Contact 15**

https://docs.google.com/forms/d/e/1FAIpQLScdEEoKTuBt66eGN187_l-Jdkk9j-LqhEyMswSEZYtyUfn7cA/viewform

8:09 AM - **Anon 15**: 2020/2021 annual dues for **P98** received and acknowledged with thanks

8:12 AM - **Anon 15**: IMG-20210407-WA0011.jpg (file attached)

This is the account for payment of annual dues. Please send me a direct message when it is done. …. …. ACAPN

8:27 AM - **Anon 33**: 👍
