# Supplementary material for: Social presence and dynamics of group communication: An analysis of a health professionals WhatsApp group chats
Source: PLoS One. 2023 Jul 17;18(7):e0288773. doi: 10.1371/journal.pone.0288773 (PMC10351686; doi:10.1371/journal.pone.0288773)
Supplement: S2 File — (DOCX) [file pone.0288773.s002.docx]

**Appendix 1** – Excerpts on the various qualitative social presence indicators

1a. Affective responses examples -

*“*Merry Christmas* to you all from the ACAPN. May this season bring you Joy, peace, success and blessings all the days of your life!*

*“Happy new year to you all once again! ACAPN is simply the best!”*

1b. Affective (Attention) examples -

Appointments (“*The following ACAPN members have been appointed to serve in the following capacities*:.. *Congratulations to you all! … Long live ACAPN!*”).

Career promotions *(“…Waoh! Congratulations @Dr ….. Another feather to your cap!”, “…Both emerged in the top 20 highest voted among the 150 contesting university*…”).

Bereavement (*There are people whose excellent deeds continue to speak volumes even years after their death. Such is the case of this Icon of Physiotherapy profession … continue to rest in peace!*).

Promos, programmes *(“We hereby call for submission of abstracts for ….”, “This is an important announcement! ….”, “Our Ordinary General Meeting (OGM) is…”*), (“*Call for submission of Articles … *ACAPN wants to launch…”*).

Clarification on issues (“*If you want to registered for WCPT conference, please do not register as…”, “…Concerning the election, *the system is online voting and there will be …*”) .

1c. Cohesive response examples -

*“Congratulations to us!”, “…this is good for us!”, “Together we shall all move higher…”, “I trust we are all keeping safe”….”We, the NEC, will continue to serve you and will never take your confidence in us for granted”.*

1d. Cohesive (Vocative) examples -

Phrases used were “*Highly esteemed members of ACAPN”; “A big congratulation to you Dr. A…. for your new achievement”. “Thanks Z…”, “Well done to …. State ACAPN”, “2021 annual dues for Dr T… received and acknowledged with thanks” and “Dr O … fondly called X … has yet again made us by proud by this beautiful presentation delivered to* ….”.

(NB: Initials used here are not of real names)

1e. Cohesive (Phatic or salutation)

These greetings include *“Hello”, Hi dear Colleagues”, “Good evening everyone”, “Long live ACAPN!”, Long live Physiotherapy Profession in Nigeria!”, “Thank you Sir for sharing”, …”Super congratulations to you Sir.”.*

1f. Interaction (complementing and expressing appreciation) examples -

*“This is great. Well done my Supervisor!”*

*“Great! Our own person is in the inner Caucus”.*

1g. Interactive (asking question) such as -

*“Any advice or suggestions on how to go about registration for WCPT please?”*

1h. Interactive (referring explicitly to others messages) examples -

mostly by saying “*congratulations*” Likewise, season greetings were responded to in typical manners, as “*wish you same, my boss*”, *yes my boss*” Some other times, responses were made to post where clarifications were deemed necessary - .

*“…you're very right sir…”, “. … I know the NEC will do the needful soon …”*

1i. Interactive (expressing agreement) –

Such as saying, “*this is a great read*” to acknowledge and express agreement to the work of others, “*Ok, good*” to express agreement to the opinions of others, and “*Great job*” to commend and express agreement to the work of others.
