# Supplementary material for: Social presence and dynamics of group communication: An analysis of a health professionals WhatsApp group chats
Source: PLoS One. 2023 Jul 17;18(7):e0288773. doi: 10.1371/journal.pone.0288773 (PMC10351686; doi:10.1371/journal.pone.0288773)
Supplement: Attachment — Submitted filename: Response to reviewers - PLOSONE - WhatsApp Study 2.docx [file pone.0288773.s003.docx]

PONE-D-22-30977

Social Presence and Dynamics of Group Communication: An Analysis of a Health Professionals WhatsApp Group Chats

Dear Editor,

Thank you for your careful consideration and review of our manuscript. Please find below the point-by-point responses to the comments.

| Editor | Comment | Response |
| --- | --- | --- |
|  | 3. We note that you have indicated that data from this study are available upon request. PLOS only allows data to be available upon request if there are legal or ethical restrictions on sharing data publicly. For more information on unacceptable data access restrictions, please see http://journals.plos.org/plosone/s/data-availability#loc-unacceptable-data-access-restrictions.  In your revised cover letter, please address the following prompts:  a) If there are ethical or legal restrictions on sharing a de-identified data set, please explain them in detail (e.g., data contain potentially sensitive information, data are owned by a third-party organization, etc.) and who has imposed them (e.g., an ethics committee). Please also provide contact information for a data access committee, ethics committee, or other institutional body to which data requests may be sent.  b) If there are no restrictions, please upload the minimal anonymized data set necessary to replicate your study findings as either Supporting Information files or to a stable, public repository and provide us with the relevant URLs, DOIs, or accession numbers. For a list of acceptable repositories, please see http://journals.plos.org/plosone/s/data-availability#loc-recommended-repositories. | This has been done |
|  | 4. Please ensure that you refer to Figures 2, 5, 6 and 7 in your text as, if accepted, production will need this reference to link the reader to the figures. | This has been done. All figures are referred to. Figures 1-4 are now merged as one. |
|  | 5. We note you have included a table to which you do not refer in the text of your manuscript. Please ensure that you refer to Table 1 in your text; if accepted, production will need this reference to link the reader to the Table. | This has been done |
| Reviewer #1 | 1. Abstract: Please provide keywords in accordance with the authors' guidelines. | This has been done |
|  | 2. Methods: insufficient. Define all the concepts you will develop in the results section (interactive / cohesive / affective com.) and qualitative concepts (Affective (expression of emotions...)) with examples. | This has been done |
|  | 3. Results: must contain the results and only the results. | This has been done |
|  | 4. Discussion: Should begin with an answer to the research question. Then discuss the weaknesses and strengths. | This has been done |
|  | 5. The verb tense should be past tense for all sections except the introduction (literature review). | This has been done |
|  | 6. All figures should be listed in the corresponding paragraph in the results section. | This has been done. |
|  | 7. Figures 1, 2, 3, and 4 may be summarized. | This has been done. |
|  | 8. Table 1, Figures 5, 6, 7 need a legend and footnote to be clearly understood. | This has been done. |
|  | 9. What does Density mean in the table? Statistical analysis It would be relevant to do a comparative analysis based on gender and trend over time. Can you perform a reproducibility test for your analysis between the 2 admin chats? | This was done and reported. The term ‘reproducibility test’ has now been used as appropriate. |
| Reviewer #2 |  |  |
|  | 1. After harvesting the chat and transferring to Gmail, who owns the data? And whose email ID was used to harvest the same? | Both ethical approval and administrative permission were obtained for this study. The data belongs to the group. OOJ email was used to harvest the chats as text files. |
|  | 2. What about the consent of other physiotherapists in the group, were their members who didn't agree for the same? | The researchers made official application for permission to conduct this study to the group. No member of the group disapproved the request. Consequently, an official signed letter to conduct study was issued by the leadership of the group. |
|  | 3. Qualitative presentation of findings: Would it look better if the examples of various elements in qualitative data are summed up in a separate appendix? This might help in 2 ways: First the look and feel of manuscript may enhance and Second this would help the future authors on the same subject to ease their manuscript without going through the pain of plagiarism correction since the examples given are such commonly used words and sentences. | An anonymized data (personal identifying information were deleted from the chats (including names, initials, gender, and phone numbers) and were coded appropriately is attached based on PLOS data availability policy.  The qualitative result section is revised. |
|  | 4. The discussion and implication of findings focuses primarily on uses of WhatsApp with its wide range of utilities in general rather than being specific about the current study. Numerous previous social studies have highlighted what has been presented in the discussion. It would be prudent to review the discussion again to make it more specific to the study actually done. | This has been done. |
|  | 5. Twice in the manuscript i could see in brackets (Error! Reference source not found), Please clarify the same. The manuscript needs a revision once again to correct few subtle mistakes | This has been done. |

All new inclusion are presented in bold format. Data have been uploaded as supplementary material.

Thank you.

Chidozie Mbada
